# Emergent polar order from interlayer microstrain in layered perovskites

**DOI:** 10.1126/sciadv.aeg3509

**Published:** 2026-08-26

**Authors:** Hangfeng Zhang, Theo Graves Saunders, Orestis Christogeorgos, Haixue Yan, Yang Hao

**Affiliations:** ^1^ School of Electronic Engineering and Computer Science, Queen Mary University of London, Mile End Road, London E1 4NS, UK.; ^2^ School of Engineering and Materials Science Queen Mary University of London, Mile End Road, London E1 4NS, UK.

## Abstract

Ferroelectric materials with switchable polarization and nonlinear dielectric responses are promising candidates for reconfigurable communication systems. However, their practical use is limited by an inherent trade-off: large dielectric tunability is usually accompanied by high dielectric permittivity and increased loss. Layered perovskite oxides such as Sr_2_Ta_2_O_7_ exhibit intrinsically low dielectric permittivity and low loss, but their centrosymmetric structure limits tunability. Here, we introduce subtle interlayer microstrain in (Ca*_x_*Sr_1-*x*_)_2_Ta_2_O_7_ via site-selective Ca substitution. The resulting microstrain gradients and asymmetric distortions of neighboring TaO_6_ octahedra break local inversion symmetry and induce dynamic polar nanoclusters within an otherwise nonpolar matrix. This configuration enables strong tunability while maintaining low dielectric permittivity and minimal loss across the broadband spectrum. The *x* = 0.08 composition shows optimal performance and enables agile frequency tuning in prototype antenna systems under applied electric fields or thermal stimulus. These findings establish interlayer microstrain engineering as a paradigm for designing high-performance, lead-free tunable microwave dielectrics for adaptive communication technologies.

## INTRODUCTION

Emergent polarization in materials that are nominally nonpolar is expanding the possibilities for designing functional dielectrics (*1*–*3*). In the centrosymmetric crystal structure, local lattice distortions, symmetry frustration, or strain gradients can give rise to hidden polar nanoclusters (PNCs) (*4*), which are localized regions of broken symmetry that exhibit nonlinear polarization responses without forming long-range polar order (*5*–*8*). These effects broaden the concept of polar instabilities and provide approaches to creating responsive dielectrics beyond those found in classical ferroelectric (FE) materials (*9*, *10*). At the same time, the growing demand for adaptive and energy-efficient electronic systems, such as agile communication hardware and next-generation sensors, has increased the need for materials whose dielectric properties can be reversibly tuned by external stimuli (*11*–*15*). Ferroelectric materials, which possess switchable polarization and nonlinear dielectric responses, are promising candidates for such reconfigurable components (*16*–*18*). However, traditional perovskite ferroelectrics, such as Ba_1-*x*_Sr*_x_*TiO_3_ (BST) and related solid solutions typically exhibit very high dielectric permittivity (ɛ’ > 2000) and significant dielectric loss (*18*–*22*), which can cause impedance mismatches and limit bandwidth in microwave circuits (*16*, *23*). The persistent trade-off between tunability, permittivity and loss has been a major challenge, motivating the search for alternative design strategies that can decouple these competing properties.

**Fig. 1.**
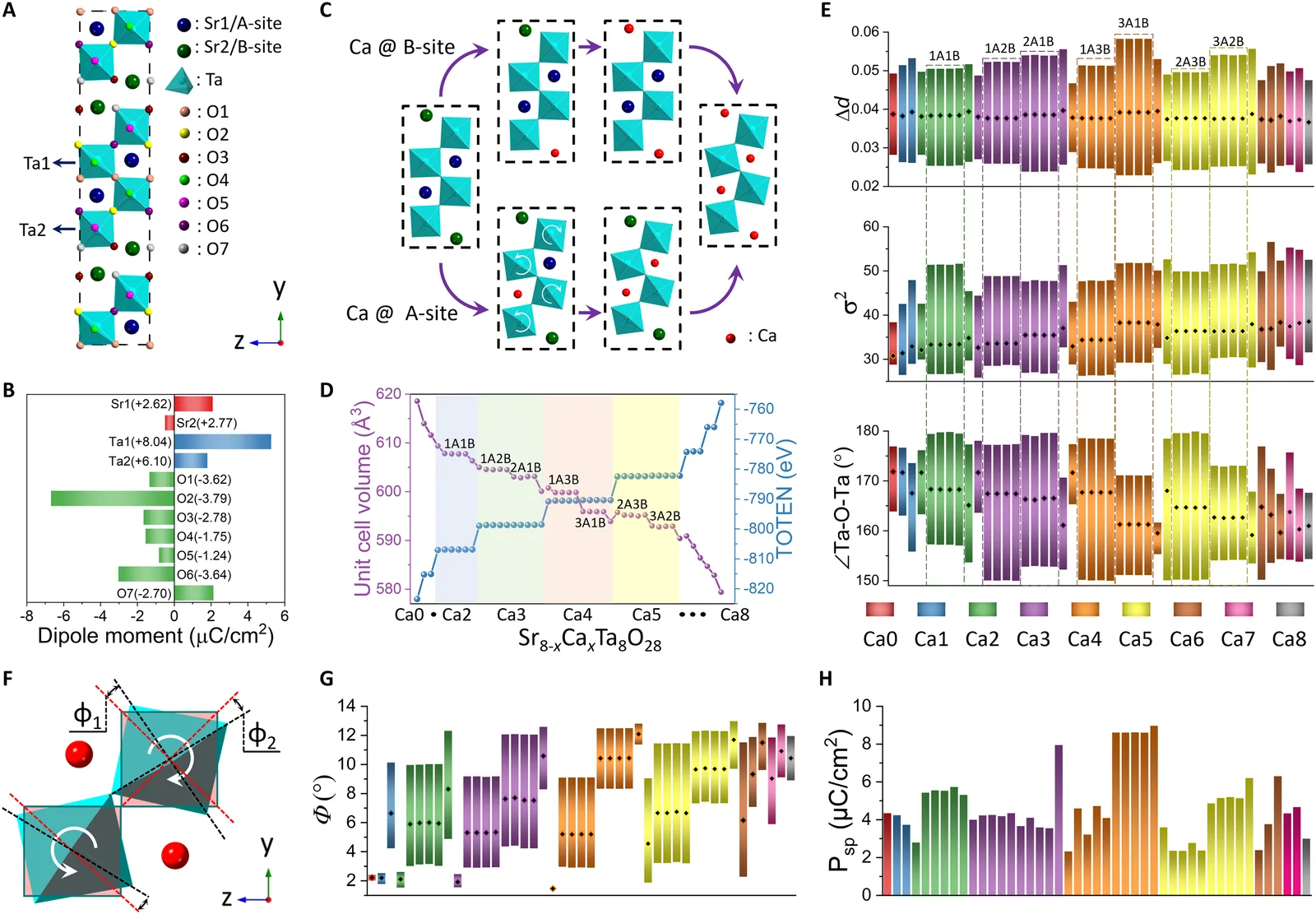
DFT-driven prediction of structure and polarization behavior in Ca-substituted Sr_2_Ta_2_O_7_. (**A**) Relaxed crystal structure of Sr_2_Ta_2_O_7_ composition along the *a*-axis within one unit cell (space group *Cmc*2_1_). (**B**) Dipole moment of ions along the *z*-axis within the unit cell, with the corresponding averaged Born effective charges given in the parentheses. (**C**) Evolution of the layered structure upon Ca substitution at different Sr sites. (**D**) Compositional dependence of the unit cell volume and total free energy (TOTEN) in Sr_8-*x*_Ca*_x_*Ta_8_O_28_ (Ca*x*) system. (**E**) Configurational dependence of TaO_6_ octahedral distortions for different Ca substitutions. (**F**) Glazer rotation angle extracted from octahedral tilts. Configurational dependence of (**G**) Glazer rotation angle and (**H**) spontaneous polarization along the *z* axis within unit cell. All structures were fully relaxed using DFT calculations. The notation *n*A*m*B denotes the configurations where *n* and *m* Ca atoms occupy the A-site (Sr1) and B-site (Sr2) positions, respectively. Diamond symbols in (E) and (G) represent the average values.

Perovskite layered structure (PLS) ferroelectrics, with the general formula M_2_M’_2_O_7_, feature a quasi two-dimensional architecture composed of four-layer slabs of corner-sharing oxygen octahedra separated by interlayers (Fig. 1A) (*24*–*26*). These materials exhibit intrinsic anisotropy, low concentrations of oxygen vacancies, and consequently low dielectric permittivity and loss (*27*, *28*). The presence of decoupled interlayers and anisotropic bonding also promotes symmetry-lowering distortions (*29*, *30*). However, most four-layer PLS ferroelectrics have very high Curie temperatures (*T*_c_ > 1000°C) (*31*, *32*), which stabilize their polar distortions and require large electric fields for reorientation. An important exception is strontium tantalum oxide (Sr_2_Ta_2_O_7_, STO), which remains centrosymmetric (paraelectric phase) at room temperature (RT) and has a much lower *T*_c_ (*ca.* –107°C) (*24*, *25*), making it a promising candidate for tunable dielectrics. Previous attempts to induce tunability in STO-based PLS systems have mainly focused on substituting its tantalum sites (e.g. Nb^5+^ doping) to modify the connectivity of octahedral network (*26*, *27*, *33*).

Here, we introduce an alternative approach based on interlayer microstrain engineering, achieved by partially substituting Sr^2+^ ions in the interlayer site with smaller Ca^2+^ ions. This targeted chemical substitution creates local lattice distortions that break inversion symmetry at the nanoscale, leading to the formation of dynamic polar nanoclusters within an otherwise centrosymmetric matrix. These PNCs can rapidly and reversibly reorient in response to external electric fields or temperature changes, resulting in strong dielectric tunability while maintaining low dielectric permittivity and loss. By employing Ca-substituted STO as a model system, we reveal that microstrain induced local symmetry frustration enables tunable polarization in the PLS system without causing high loss and high permittivity. We further validate the functional potential of this concept by integrating the optimized ceramics into prototype microwave resonators and antennas. Our results establish interlayer microstrain engineering as an effective strategy for dynamic polarization control in PLS ferroelectrics, connecting emergent polarization phenomena with next-generation tunable dielectric technologies.

## RESULTS

### Density functional theory (DFT) simulations of site-specific structural evolution

The parent Sr_2_Ta_2_O_7_ ferroelectric model was constructed using the reported polar *Cmc*2_1_ structure as a reference for evaluating local polarization tendencies. This structure contains two distinct Sr sites, two Ta sites, and seven oxygen sites, each occupying four equivalent positions (Fig. 1A). DFT relaxation of this model reveals large Born effective charges on Ta (balanced by oxygen), which indicate a strong second-order Jahn-Teller off-centering effect arising from Ta-O hybridization (Fig. 1B). (*34*–*36*) This effect leads to pronounced spontaneous polarization, primarily dominated by oxygen displacements within the TaO_6_ octahedra, which contribute nearly twice as much as the Ta ions and five times as much as the Sr ions. To investigate the effects of chemical substitution, Ca^2+^ ions were introduced at either the perovskite layer A-site (Sr1) or interlayer B-site (Sr2), simulating a range of configurations (fig. S1 and table S1). Because Ca^2+^ has a smaller ionic radius than Sr^2+^ (*37*), its substitution modifies the Ta-O bond network and reduces the interlayer spacing, resulting in significant distortion and tilting of the TaO_6_ octahedra, as well as enhanced oxygen displacements (Fig. 1C). These local structural changes decrease the unit cell volume and increase total free energy (TOTEN) (Fig. 1D). In particular, configurations with a higher proportion of Ca at the A-site (perovskite layer) show even greater reductions in unit cell volume due to intense local lattice compression. While the average Ta-O bond length distortion index (∆*d*) does not change significantly with composition, its distribution becomes broader at intermediate Ca concentrations, especially in A-site rich models (Fig. 1E). Similarly, the variance in O-Ta-O bond angles (σ^2^) increases with A-site substitution, indicating greater octahedral distortion. A-site substitution also causes a larger reduction in the average Ta-O-Ta bond angle, indicating more severe disruption of the octahedral tilting. The octahedral tilting, quantified by the Glazer rotation angle (Φ) along *x*-axis, is enhanced by A-site Ca substitution (both clockwise and counterclockwise) compared to B-site substitution. Although these competing tilt patterns may compensate for each other structurally, they influence the net spontaneous polarization depending on the spatial arrangement and concentration of Ca.

### Composition-dependent phase evolution and dielectric response

**Fig. 2.**
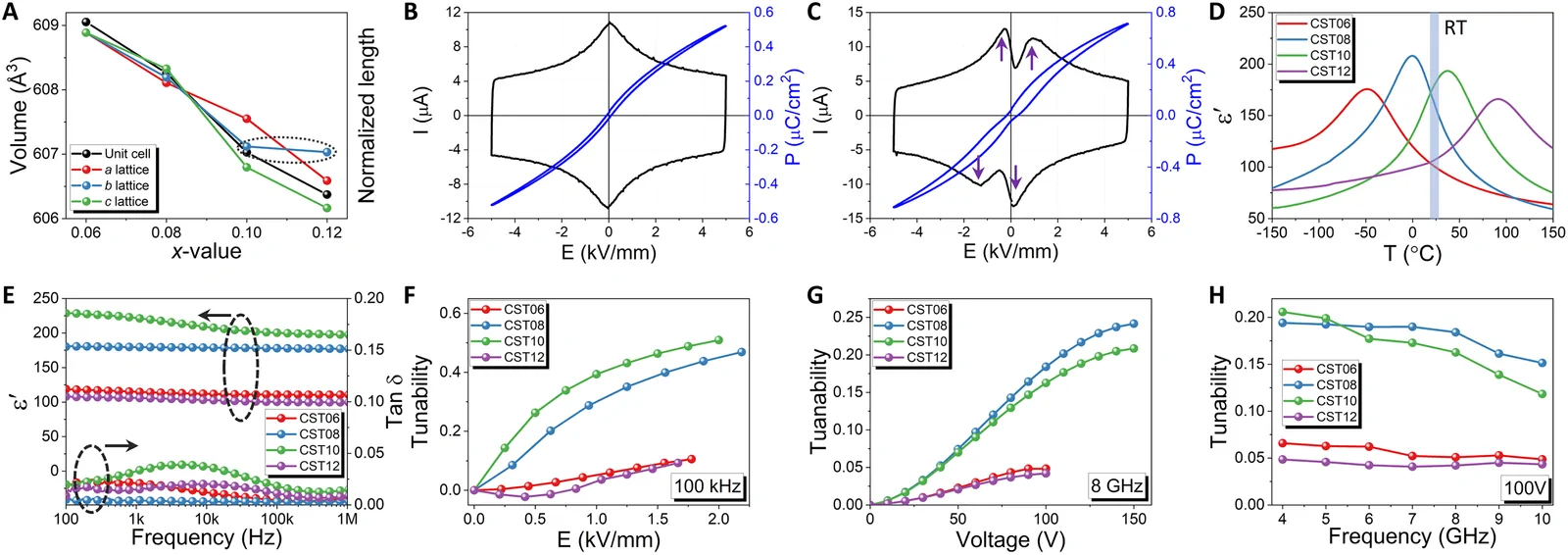
Crystal structure, dielectric, ferroelectric, and tunability behavior of CST ceramics. (**A**) Unit cell volume and normalized lattice parameters for the studied compositions. *I–E*/*P–E* loops measured at 10 Hz for (**B**) CST08 and (**C**) CST12. (**D**) Temperature dependence of dielectric permittivity for the studied compositions. (**E**) Frequency dependence of dielectric properties for studied compositions. (**F**) Electric field dependence of dielectric tunability for studied compositions, measured at 100 kHz. (**G**) Voltage dependence of dielectric tunability for studied compositions at 8 GHz and (**H**) frequency dependence of microwave tunability measured at 100 V for studied compositions.

Complementing these theoretical predictions, experimental results on (Ca*_x_*Sr_1-*x*_)_2_Ta_2_O_7_ ceramics (*x* = 0.06–0.12; CST06-CST12) reveal a composition-dependent structural evolution. XRD patterns were well fitted using PLS models with space groups *Cmcm* (*x* = 0.06–0.10) and *Cmc*2_1_ (*x* = 0.12) (fig. S2 and table S2). SEM images on the surface reveal dense ceramics (>95% theoretical density) with tightly packed, platelet-like grains consistent with the anisotropic PLS framework. As Ca content increases, lattice length *a*, *c* and unit cell volume show a general decrease (Fig. 2A), in agreement with the trend in DFT. The *b* lattice parameter, which reflects the thickness of the perovskite slab and the spacing between interlayers, generally decreased with increasing Ca content but showed an unexpectedly small reduction at *x* = 0.12. This anomaly may be related to the changes in interlayer spacing and the preferential occupation of specific lattice sites by Ca. (*28*) These observed structural changes are consistent with the distortion mechanisms and site preference trends predicted by DFT, where local structural disorder can lead to macroscopic symmetry breaking.

Compositions CST06 and CST08 show narrow hysteresis *P-E* loops with two switching currents near zero field in the *I-E* loops (Fig. 2B and fig. S3), consistent with randomly orientated polar nanoclusters exhibiting reversible dipolar switching. (*18*) With increasing Ca content, saturation polarization increases and current peaks sharpen, indicating partial alignment of polar clusters. The slightly shifted current peaks and weak remanent polarization in CST10 further suggest the existence of FE domains. Remarkably, CST12 exhibits antiferroelectric-like (AFE) behaviour, with a double hysteresis *P-E* loop and four current peaks in *I-E* loop (Fig. 2C), attributed to a field-induced phase transition between weakly and strongly polar states (*28*). To evaluate whether these polar states can be stable after poling, room-temperature *d*_33_ values were measured after electrical poling at 2 kV mm^−1^. CST06, CST08, and CST10 show negligible *d*_33_, while CST12 shows only a weak response of approximately 1 pC N^−1^. These results are consistent with the small remanent polarization and indicate that the field-induced polar response is largely reversible. The temperature dependence of dielectric permittivity (ɛ’) and loss tangent (tanδ) for the studied compositions shows a frequency-independent Curie point (*T*_c_) (fig. S4), corresponding to the ferroelectric-paraelectric phase transition. The *T*_c_ increases with Ca concentration (Fig. 2D), indicating that local distortions induced by Ca substitution progressively strengthen the polar order. Modified Curie-Weiss analysis on high-temperature dielectric spectra (above *T*_c_) yields dielectric diffuseness exponents (γ) ranging from 1.5 to 1.7 (table S3), indicative of intermediate diffuseness behaviour. The γ value reflects the distribution of local polar environments and their thermal response. Thus, the relatively high γ of CST10 is consistent with its pronounced frequency dispersion in ε’ near room temperature (Fig. 2E), suggesting the presence of active FE domains that become immobilized at higher frequencies. In contrast, although CST12 has a higher *T*_c_, its lower γ indicates a less diffuse transition, consistent with its antiferroelectric-like response. CST06, CST08 and CST12 show constant ε’ across the measured frequency range. Notably, CST08 exhibits the lowest dielectric loss, which may be related to its low *T*_c_ (below RT) and minimal defect concentration. Importantly, all CST samples have low dielectric permittivity (ε_max_′ < 250), more than an order of magnitude lower than that of typical BST-based tunable ferroelectrics. This low permittivity is advantageous for microwave communication applications as it reduces impedance mismatch between the dielectric component and free space (or feedlines), thereby enabling broader bandwidths and improved signal integrity (*38*). DFT simulations corroborate this mechanism, showing that high Ca substitution disrupts the TaO_6_ rotation, leading to opposite tilts of adjacent octahedra and the formation of an antipolar structure. Under an applied electric field, this antipolar arrangement can be realigned into a strong polar state and reverse back after the field is removed, producing the observed double hysteresis loop. Upon heating, these current peaks shift toward zero field and merge at 100°C (fig. S5), marking the transition to a paraelectric state.

Dielectric tunability (η), defined as the relative change in ε′ under a DC bias, was calculated by using

$$\eta =\frac{\varepsilon_{0}^{\prime }-\varepsilon_{E}^{\prime }}{\varepsilon_{0}^{\prime }}$$

where ε_0_’ and ε*_E_*’ are the dielectric permittivity at zero and applied electric fields, respectively. At 100 kHz, CST06 exhibits a gradual, linear increase in *η* (Fig. 2F), consistent with a low density of polar nanoclusters. In contrast, CST08 and CST10 show sharp increases in *η* at low electric field (<1 kV mm^−1^), followed by saturation behaviour at high fields, suggesting rapid dipole alignment and restricted dipolar motion under bias. Notably, CST12 exhibits negative tunability below *ca*. 0.7 kV mm^−1^, where ε′ increases with field. This anomalous behaviour is likely associated with a field-induced phase transition, consistent with the double hysteresis *P-E* loop. The tunability behaviours of CST06-CST10 compositions are correlated with the concentration and dynamics of the polar nanoclusters or FE domains, which vary with Ca substitution. CST06 has low permittivity and low loss but limited tunability, while CST12 has low permittivity but shows anomalous low field tunability. CST10 exhibits higher permittivity and stronger low frequency tunability but also shows pronounced frequency dispersion and higher dielectric loss. By contrast, CST08 combines relatively low permittivity, low dielectric loss, weak frequency dispersion, and strong field tunability.

At microwave frequencies (4 to 10 GHz), CST06 and CST12 exhibit low and nearly frequency-independent ε′ (fig. S6), which is attributed to the short relaxation time in CST06 and the antipolar structure in CST12. In contrast, CST08 and CST10 show a frequency-dependent decrease in ε′. CST08 achieves the highest dielectric permittivity (153 at 4 GHz), with only minor reduction from its low frequency value (177 at 1 MHz) and maintains stable permittivity up to 8 GHz. CST10, although it has a higher ε′ at low frequency, experiences a steeper decline at high frequency, likely due to the presence of larger and less dynamic ferroelectric domains. At 1.68 GHz, CST08 exhibits real and imaginary dielectric permittivity values of 172.7 and 1.6, respectively, corresponding to a very low loss tangent of 0.9% as measured by the resonant cavity method. Under DC bias, all compositions show decreasing permittivity with increasing frequency (fig. S6), which is due to dipole alignment and suppression of dipolar oscillations. At 8 GHz, CST08 demonstrates superior permittivity and tunability compared to CST10 (Fig. 2G), achieving a maximum tunability of 24% at 150 V. While CST10 outperforms CST08 in tunability below 5 GHz, it suffers a sharper performance drop at higher frequencies (Fig. 2H), supporting the conclusion that large ferroelectric domains in CST10 contribute less at high frequencies. In contrast, the presence of small PNCs in CST08 is associated with stable polarization dynamics across a broad frequency range, resulting in enhanced tunability and low-loss response (*39*). Compared with representative tunable dielectrics, CST08 exhibits a rare combination of high microwave tunability, low dielectric permittivity, and low loss, demonstrating the advantage of interlayer microstrain engineering for tunable microwave applications (table S4).

### Local structural characterization

**Fig. 3.**
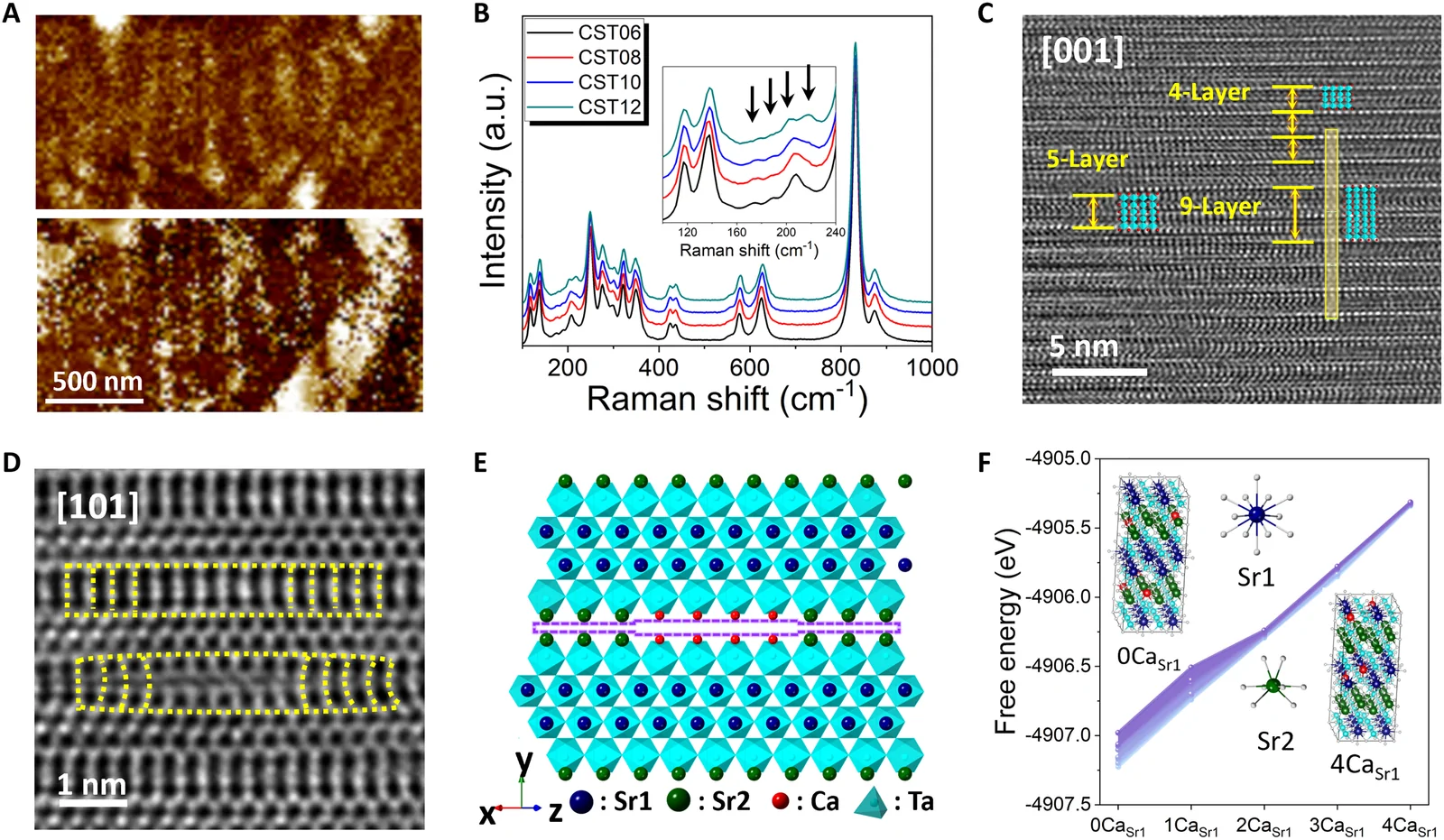
Nanoscale evidence of polar nanoclusters and microstrain-induced symmetry breaking. (**A**) PFM amplitude (upper) and phase (lower) images of CST08; (**B**) Raman spectra of studied compositions with enlarged view for specific phonon modes; (**C**) HRTEM image along the [001] direction with 4-, 5- and 9-layer stacking sequences; (**D**) HRTEM image of defect region. (**E**) Atomic model of PLS structure representing the expanded interlayer spacing by Ca substitution. (**F**) Calculated Helmholtz free energy for Sr_44_Ca_4_Ta_48_O_164_ models as a function of Ca site occupancy. (Inset) Crystal structure of 3 × 1 × 2 supercells with 4 Ca randomly occupied in 48 Sr sites. Schematic of Sr-O bonding with two configurations, Sr1: CN = 12 and Sr2: CN = 8.

Piezoresponse force microscopy (PFM) was employed to investigate the evolution of the polar structures in CST08-CST12 compositions (Fig. 3A and fig. S7). The topographic images reveal smooth ceramic surfaces with root-mean-square roughness (R_q_) of below 2.5 nm, effectively minimizing topography-induced artefacts in the PFM measurement. In CST08, the amplitude and phase images showed weak contrast with nanoscale stripe patterns, indicating the presence of small, randomly orientated polar nanoclusters embedded in a nonpolar matrix. In CST10, pronounced stripe patterns emerge, consistent with the formation of large FE domains. Interestingly, this domain growth did not persist in CST12, where the PFM images show disrupted and fragmented FE domain features. This disruption in domain configuration at higher Ca concentrations is attributed to the emergence of an incommensurate antipolar structure, as predicted by DFT calculations and supported by the AFE-like double hysteresis observed in the *P-E* loop. This increased local disorder further suppresses long-range polar ordering, leading to fragmented and unstable FE domain structures.

Raman spectra of the studied compositions reveal multiple vibrational modes below 1000 cm^−1^ (Fig. 3B and fig. S8), consistent with the PLS system (*40*). The low-wavenumber modes (<300 cm^−1^), primarily attributed to Sr/Ca vibrations, exhibit notable shifts, while the high-wavenumber modes (500–600 cm^−1^), associated with octahedral tilting, shift to higher wavenumbers with increasing Ca content, indicating changes in local distortions. Furthermore, the observed peak splitting in the 160–240 cm^−1^ region confirms local symmetry breaking and the formation of polar structures. TEM image of CST08 showed platelet grains with stripe-like features (fig. S9A), consistent with PFM observations. Selected area electron diffraction (SAED) along [010] zone axis revealed extra superlattice reflections, attributed to incommensurate phase modulations and polar nanocluster formation (fig. S9B) (*25*, *27*, *41*). High-resolution TEM (HRTEM) along [001] reveals a variable layer-stacking sequence consisting of 4-, 5- and 9-layer perovskite slabs (Fig. 3C). Line profile analysis confirms peak-to-peak distances of *ca.* 1.3 nm (4-layer) and 2.9 nm (9-layer), indicative of stacking faults (fig. S9D). These structural variations help reduce oxygen vacancy concentrations, a common defect induced by elevated temperature sintering, which increases dielectric loss. The incorporation of stacking faults disrupts the oxygen sublattice, which could mitigate defect formation and improve dielectric properties. (*27*) Additional HRTEM along [101] zone axis reveals mostly 4-layer stacking but also some locally disordered atomic arrangements (fig. S9, E and F). Closer inspection of these anomalies reveals enlarged interlayer gaps, indicative of microstrain induced by ionic size difference between Ca^2+^ and Sr^2+^ (Fig. 3D). This microstrain locally creates a spacing gradient along the interlayer (Fig. 3E), which is associated with disrupting the nonpolar matrix and the presence of polar nanoclusters. This represents the first direct atomic-scale observation of microstrain-driven polar nanocluster formation in the PLS system.

To investigate the site preference of Ca substitution in CST08, a 3 × 1 × 2 supercell model containing 48 Sr positions was constructed (Fig. 3F). Four Ca^2+^ ions were introduced per model, corresponding to the composition (Ca_0.083_Sr_0.917_)_2_Ta_2_O_7_, closely matching CST08 stoichiometry. Five substitution schemes were designed by fixing the total Ca content to four atoms and varying their distribution between the Sr1 and Sr2 sites. In model 0Ca_Sr1_, all Ca atoms occupied Sr2 sites; in 4Ca_Sr1_ model, all Ca atoms were placed on Sr1 sites, with intermediate configurations (1Ca_Sr1_, 2Ca_Sr1_, 3Ca_Sr1_) representing mixed distributions. For each scheme, ten randomly generated configurations were fully relaxed using DFT, yielding fifty models for comparative Helmholtz free energy analysis. The 0Ca_Sr1_ and 4Ca_Sr1_ models exhibited the lowest (−4911.26 eV) and highest (−4909.59 eV) free energy, respectively, indicating a thermodynamic preference for Ca substitution at interlayer sites. Models with higher Sr1 site occupancy show narrow energy dispersion, suggesting energetic homogeneity among Sr1 sites, while greater variation among Sr2-rich models indicates more sensitivity to the arrangement of Ca sites. The smaller ionic radius of Ca^2+^ is better accommodated by the spatially constrained 8-fold coordinated Sr2, introducing localized lattice distortions and interlayer microstrain that disrupt the centrosymmetric matrix and stabilize polar nanoclusters. Combined with the DFT results, which show that Ca substitution modifies the Ta-O framework and induces asymmetric distortion/tilting of neighbouring TaO_6_ octahedra, these observations suggest that interlayer microstrain locally disrupts the centrosymmetric matrix. With increasing Ca content, the local polar correlations become progressively strengthened, evolving from weak nanoscale PNC features in CST08 to more pronounced domain-like polar contrast in CST10. This local symmetry breaking is consistent with the stripe-like TEM contrast, Raman peak splitting, and PFM response observed in CST08, supporting the formation of polar nanoclusters within the nonpolar layered framework.

### Field-driven frequency modulation enabled by tunable layered dielectrics

**Fig. 4.**
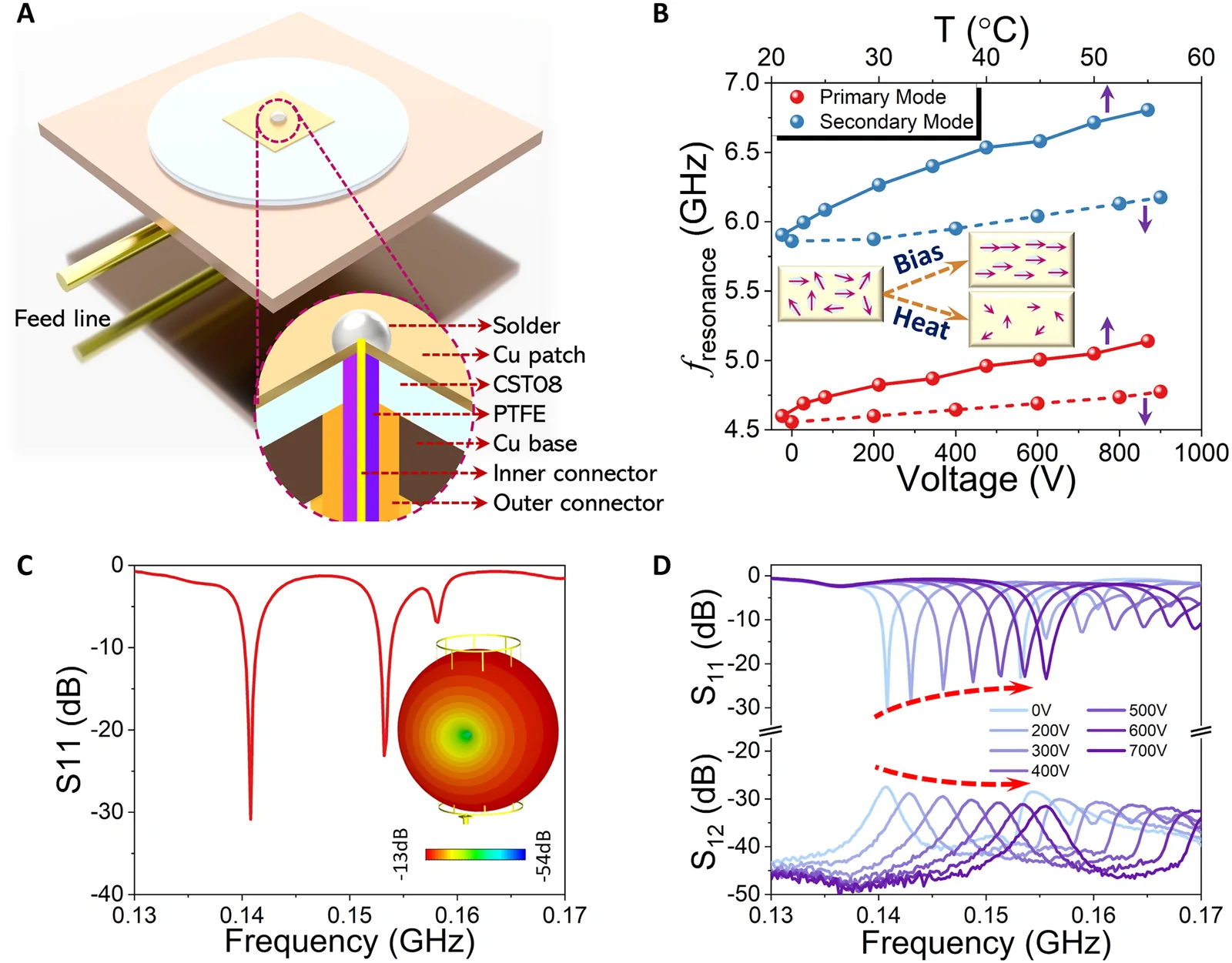
Voltage and temperature-controlled frequency agility in CST08-based resonators. (**A**) Schematic illustration of the CST08-based patch antenna. The device consists of a copper base plate of 35 × 50 × 3 mm^3^, a CST08 ceramic disk with a diameter of *ca.* 26 mm and thickness of 1.5 mm, and a top copper patch of 12 × 14 mm^2^. A central hole of approximately 1.5 mm was drilled through the ceramic disk to accommodate the SMA feed, with an outer conductor diameter of 2 mm; (**B**) DC bias voltage and temperature dependence of resonance frequency for primary and secondary modes; (**C**) Measured reflection coefficient spectrum S_11_ and simulated 3D radiation pattern of a low-pass birdcage resonator integrating CST08 ceramics as capacitive elements at each arm midpoint; (**D**) Reflection spectra S_11_ and transmission spectra S_12_ vary with applied bias voltage.

To verify that microstrain induced dielectric tunability in CST08 enables functional frequency agility, we integrated the ceramics into two RF resonator architectures representative of practical communication platforms. A voltage and thermally tunable microwave resonator was constructed by incorporating CST08 as the active dielectric layer, sandwiched between a copper ground plane and patch, and excited via a centre-fed coaxial probe (Fig. 4A). Under applied DC bias via a bias tee, the S_21_ transmission spectrum exhibits two main resonant notches that shifted towards higher frequency with increasing voltage (fig. S10). The primary mode shifted from 4.56 to 4.77 GHz and the secondary mode from 5.87 to 6.18 GHz, with a near-linear voltage dependence (Fig. 4B), attributed to field-induced suppression of PNC vibrations and a corresponding reduction in dielectric permittivity. Thermal modulation was also explored between 21°C and 55°C (fig. S10). As the temperature increased, both primary peak and secondary peak shifted from 4.60 to 5.14 GHz and from 5.91 to 6.81 GHz, respectively, indicating a thermally driven decrease in permittivity due to a reduced density of PNCs. These results demonstrate that dielectric tunability in CST08 can be functionally accessed through both electrical and thermal stimuli in real device geometries, without relying on external varactors or circuit reconfiguration.

An electrically small birdcage antenna was designed and fabricated using CST08 as tunable capacitive elements (fig. S11). This low-pass configuration consists of two conductive rings (driven and floating), connected by symmetrically spaced arms, each containing a CST08 capacitor at its midpoint. This structure induces propagation delay in the RF current path, generating sinusoidal, phase-shifted currents between adjacent arms. The low-pass design enables direct DC biasing across the rings, eliminating the need for a bias tee and preventing high voltage backflow to the feed line. The 3D radiation pattern (Fig. 4C) is nearly omnidirectional with a maximum gain of −13 dBi, and the 2D cuts at φ = 0° and φ = 90° confirm symmetric two-lobed patterns along orthogonal planes (fig. S12). As the DC bias increases, the resonance frequency of the antenna, observed in both S_11_ and S_12_ spectra, shifts from 0.14 GHz (0 V) to 0.16 GHz (700 V) (Fig. 4D). The birdcage design produces a highly uniform, rotating magnetic field, which is ideal for applications such as helicon-plasma generation and magnetic resonance imaging (MRI). In this context, its narrowband, quasi-omnidirectional response provides a robust platform for demonstrating materials-based frequency agility in compact RF environments. By replacing conventional fixed capacitors with tunable CST08 ceramics, the system achieves real-time, field-driven frequency control, demonstrating that engineered polar nanocluster dynamics can enable rapid and adaptive electromagnetic responses. To examine the bias-on/bias-off reproducibility, the *S*_11_ spectrum of the birdcage resonator was measured sequentially at 0 V and under increasing DC bias from 200 to 700 V, with the bias removed and the 0 V spectrum re-measured after each voltage step (fig. S13). The resonance shifts systematically toward higher frequency under applied bias and returns close to its initial 0 V position after field removal, confirming reversible voltage-controlled frequency tuning at the device level. This device-level reversibility is consistent with the largely recoverable field-induced permittivity and polarization response observed in the dielectric and ferroelectric measurements, supporting the role of reversible polar nanocluster dynamics in CST08. These device-level results confirm that interlayer microstrain engineered PNCs offer a viable approach for agile frequency modulation across different RF domains, thereby validating the design principle from atomic structure to system performance.

## DISCUSSION

This work demonstrates that interlayer microstrain engineering offers a powerful strategy to control polarization and achieve low-loss dielectric tunability in layered perovskites. In (Ca*_x_*Sr_1-*x*_)_2_Ta_2_O_7_, site-selective Ca substitution at interlayer Sr sites induces localized lattice compression and asymmetric distortion and tilting of TaO_6_ octahedra, breaking inversion symmetry and forming dynamic polar nanoclusters (PNCs) within a nominally centrosymmetric lattice. These PNCs exhibit a rapid and reversible polarization response, enabling low dielectric loss and high tunability across a broad frequency range. At higher Ca content (*x* = 0.12), the system exhibits an antiferroelectric-like behaviour with a double hysteresis *P-E* loop, consistent with the DFT relaxed structure showing antiphase rotations of adjacent TaO_6_ octahedra that favour antipolar coupling. Integration of optimal composition *x* = 0.08 into microstrip and birdcage RF resonators confirms real-time, voltage- and temperature-controlled frequency agility. Together, these results demonstrate that microstrain-induced polar nanoclusters can enable tunable dielectric properties in (Ca*_x_*Sr_1-*x*_)_2_Ta_2_O_7_, with potential implications for adaptive communication systems.

## MATERIALS AND METHODS

### Materials preparation

(Ca*_x_*Sr_1-x_)_2_Ta_2_O_7_ (CST100*x*, *x* = 0.06, 0.08, 0.10 and 0.12) compositions were synthesized using the solid-state method. The stoichiometric amounts of the starting materials, calcium carbonate (CaCO_3_, 99.5%, Sigma-Aldrich), strontium carbonate (SrCO_3_, 99.9%, Sigma-Aldrich) and tantalum oxide (Ta_2_O_5_, 99.99%, Sigma-Aldrich) were mixed with zirconia balls within a nylon jar filled with ethanol. Subsequently, a ball milling procedure was conducted in a planetary ball mill machine (PULVERISETTE 5/4 classic line, Germany) for a duration of 20 hours at a speed of 180 rpm. The resulting slurry was then dried in an oven at 100°C and subsequently sieved through a 250 μm sieve. The obtained powder underwent calcination at 1200°C for 4 hours, followed by a second ball milling process, drying, and sieving. The resulting powder was pressed into discs using a uniaxial pressure of 250 MPa and sintered at 1600°C for 2 hours. The density of the sintered pellets was measured using the Archimedes method in water.

### Materials characterization

The studied ceramics were crushed and ground into fine powder for x-ray powder diffraction (XRD) measurement. A PANalytical CUBIX3 diffractometer equipped with an X’Celerator detector and utilizing Ni filtered Cu-Kα radiation (λ = 1.5418 Å) was employed. The diffraction data was recorded at room temperature, employing a flat plate Bragg-Brentano geometry over the 2θ range of 5–120°, with a step width of 0.0334° and each step had an effective count time of 200 s. Subsequently, the obtained data was analyzed using the GSAS suite program through Rietveld analysis. (*42*) Initially, an orthorhombic structure with the *Cmcm* space group was selected as the initial model for fitting the XRD patterns (*26*). The morphology of the ceramics was examined by scanning electron microscopy (SEM) using an FEI Inspect F instrument. Piezoresponse Force Microscopy (PFM) was performed utilizing an Atomic Force Microscopy (AFM) system (Bruker Dimension Icon, US) with an SCM-PIT-V2 conductive probe (Bruker, US).

### Electrical measurements

Sintered ceramics were coated with silver paste (Gwent Electronic Materials Ltd., Pontypool, U.K.) to form conductive layers for dielectric measurement. Dielectric properties at low frequency (up to 1 MHz) were measured using a precision impedance analyser (Agilent 4294A) at room temperature. Temperature-dependent dielectric properties were measured by employing an LCR meter (Agilent 4284A) and a temperature-controlled chamber. Ferroelectric measurements, including current-electric field (*I-E*) and polarization-electric field (*P-E*) were conducted using a ferroelectric hysteresis tester (NPL, UK), with voltage applied in a triangle waveform. (*43*) Microwave dielectric properties were assessed by applying a co-planar waveguide (CPW) transmission line made of gold onto the ceramics, by means of a thermal evaporator. The CST08 sample was measured using a resonant cavity method. An 80 mm diameter 2 mm thick disc was tested using a Keysight 85072A 10-GHz Split Cylinder Resonator, connected to a Keysight PNA-L running N1500A characterization software. The disc was placed between the two halves of the cavity and clamped in position using a built-in micrometer. The coupling loops were adjusted under guidance from software to compensate for loading due to a sample. Resonant peak from cavities was then used by software to calculate permittivity and loss. To evaluate dielectric tunability at microwave frequencies, the propagation constant was deduced from 2-port S-parameter measurements conducted with a PNA-L N5230C Vector Network Analyzer, featuring full calibration with a GSG microprobe 525. The transmission line was a 5 mm long silver layer. The CST08 disk used in the patch antenna was polished on both surfaces. A central hole was drilled through the disk. A rigid coaxial feed cable, stripped so its PTFE dielectric ended flush with the top surface of the disk and its shield flush with a backing copper plate, was soldered (SAC305) through a matching 3 mm aperture on that plate. The CST08 disk was then soldered to the copper plate around the via using a silver/indium alloy. Finally, a small copper top electrode (a thin square foil with a 2 mm center hole) was aligned over the protruding center conductor and soldered, completing the tunable capacitor element.

### Ab initio calculations

First-principles calculations were performed to evaluate the site preference of Ca substitution and its effect on lattice distortion using the VASP package (Vienna ab-initio simulation package) (*44*). A meta-GGA exchange-correlation functional was utilized, in particular, the recently developed r^2^SCAN function (*45*). The Perdew-Burke-Ernzerhof functional (*46*) and projector augmented wave potentials (*47*) were applied. For single unit cell structural relaxations, a plane-wave kinetic-energy cutoff of 600 eV was applied throughout. The Brillouin zone was sampled using a Γ-centered 8 × 2 × 6 Monkhorst–Pack mesh. Atomic positions and lattice vectors were fully relaxed using a conjugated gradient algorithm. Electronic self-consistency was achieved to 1 × 10^−6^ eV. All symmetry operations were disabled when treating Ca substituted configurations to avoid artificial symmetry constraints. Once relaxed, Born effective charges were obtained using DFPT in VASP. Linear-response calculations used the PBE-GGA functional and the same numerical parameters as the relaxation stage. Electronic convergence for DFPT was tightened to 1 × 10^−8^ eV to yield accurate response properties.

The crystal structure of Sr_2_Ta_2_O_7_ was taken in the orthorhombic *Cmc*2_1_ space group as the parent model. The unit cell contains 8 Sr sites, which were divided into two sets of four (Sr1: perovskite site/ A-site and Sr2: interlayer site/B-site) based on their Wyckoff positions. To model Ca substitution, configurations with *n* Ca atoms replacing Sr were generated by enumerating all possible combinations of Ca occupation on the eight Sr sublattice sites. For each configuration, a new structure was generated with appropriate site occupancies while retaining the pristine Ta-O framework. To identify the most statistically representative models of a random Sr_1-*x*_Ca*_x_* distribution, a pair-correlation based Special Quasirandom Structure (SQS) approach was employed. For each candidate configuration, site occupation variables σ*_i_* = +1 (Sr) or −1 (Ca) were assigned to the eight Sr sublattice sites. Pair-correlation functions < σ*_i_* σ*_j_* > were then computed for the first five Sr-Sr coordination shells of the parent lattice. These were compared against the ideal random-alloy target correlation value, (1-2*x*)^2^, where *x* is the Ca concentration. The root-mean square (RMS) deviation between calculated and target correlations, weighted by the number of pairs per shell, was used as a figure of merit. Configurations with the lowest RMS were identified as optimal SQS candidates. In addition, models prioritizing Ca substitution at either the Sr1(A-site) or Sr2(B-site) positions were also constructed for DFT simulation to investigate the site dependent effect of Ca substitution.

To model the CST08 composition, we constructed 3 × 1 × 2 supercells (264 atoms, with the general formula (Ca_0.083_Sr_0.917_)_2_Ta_2_O_7_) in which four Ca atoms substituted for Sr among the 48 Sr sites. Five representative substitutional configurations were defined to cover distinct site-occupancy patterns, and for each configuration ten randomly generated models were fully relaxed to obtain the total free energy. Given the large real-space cell, Brillouin-zone sampling employed a Γ-centered Monkhorst–Pack mesh of 1 × 1 × 1, along with a Gaussian smearing parameter of 0.05 eV. All simulations were carried out with a global break condition for the electronic loop of 1 × 10^−4^ eV at the gamma point of the Brillouin zone. Ionic positions were relaxed using the conjugate-gradient algorithm with the cell shape and volume fixed.

The electromagnetic performance of the birdcage antenna was simulated using CST Studio Suite (Computer Simulation Technology, Dassault Systèmes, Germany). The model consisted of a cylindrical birdcage resonator with copper conductors and a CST08 ceramic sample placed at the center. A discrete port and tuning capacitor (3.5 pF) were used to achieve impedance matching near 140 MHz. The simulations employed the frequency-domain solver with open boundary conditions and adaptive mesh refinement to ensure convergence. Far-field radiation patterns, S_11_ parameters, and 2D angular cuts were extracted to evaluate the resonance characteristics, gain distribution, and field homogeneity.

## Supplementary Materials

### This PDF file includes:

Supplementary Text Figs. S1 to S13 Tables S1 to S4 References
