# Supplementary material for: Emergent polar order from interlayer microstrain in layered perovskites
Source: Sci Adv. 2026 Aug 26;12(35):eaeg3509. doi: 10.1126/sciadv.aeg3509 (PMC13510615; doi:10.1126/sciadv.aeg3509)
Supplement: Supplementary file 1 [file sciadv.aeg3509_sm.pdf]

Science Advances
AAAS

Supplementary Materials for

Emergent polar order from interlayer microstrain in layered perovskites

Hangfeng Zhang *et al.*

Corresponding author: Yang Hao, y.hao@qmul.ac.uk

Sci. Adv. **12**, eaeg3509 (2026)
DOI: 10.1126/sciadv.aeg3509

This PDF file includes:

Supplementary Text
Figs. S1 to S13
Tables S1 to S4
References

Supplementary Text

Octahedral distortion analysis

The distortion of the TaO_6 octahedron was quantified using both bond length distortion and bond angle variance parameters. The bond length distortion parameter (Δd) (*34*) was calculated as:

$$\Delta d = \frac{1}{n}\sum_{i=1}^{n}\left(\frac{d_i - d_m}{d_m}\right)^2 \tag{1}$$

where d_i denotes each of the six individual Ta-O bond lengths and d_m is the average Ta-O bond length in the octahedron. This parameter quantifies the deviation of each bond length from the average, reflecting the degree of bond length distortion within the octahedron. Larger values of Δd indicate greater deviations from equal bond lengths in an ideal octahedron and may be associated with local strain or cation size mismatch.

The bond angle variance (σ^2) (*48*) was used to evaluate angular distortion and is defined as:

$$\sigma^2 = \frac{1}{m-1}\sum_{i=1}^{m}(\theta_i - 90°)^2 \tag{2}$$

where θ_i are the O-Ta-O angles that are ideally 90° in a perfect octahedron. For a TaO_6 octahedron, there are 15 possible O-Ta-O angles, but only the 12 cis angles between adjacent oxygen atoms, which are nominally 90°, are considered here. The three trans-angles (ideally 180°) are excluded from this calculation. The bond angle variance measures the angular distortion of the octahedron away from ideal right angle geometry, with higher values indicating greater distortion caused by octahedral tilting, rotation, or lattice strain.

Glazer tilting angle (along *x*-axis) analysis

The Glazer tilting angles were evaluated from relaxed structures obtained via DFT simulations using the four oxygen atomic coordinates within the TaO_6 octahedron. Two vectors connecting approximately opposite oxygen atoms were selected to form the octahedral diagonals, D_1 and D_2. Each diagonal vector was then projected into the *yz*-plane as:

$$D_i = (D_{iy}, D_{iz}) \tag{3}$$

A pseudocubic reference vector was defined along each diagonal direction (with the same sign and magnitude but aligned along global y or *z*-axis):

$$D_{ref} = (sgn(D_y)\cdot(|D_{1y}| + |D_{2y}|), sgn(D_z)\cdot(|D_{1z}| + |D_{2z}|)) \tag{4}$$

The tilting angle Φ_i for each diagonal is calculated as the angle between the projected diagonal and its corresponding reference axis:

$$\Phi_i = \cos^{-1}\left(\frac{D_{yz}\cdot D_{ref}}{|D_{yz}|\cdot|D_{ref}|}\right) \tag{5}$$

The Glazer tilt angle Φ for each Ta site was calculated as the mean of the two diagonal angles:

$$\Phi = \frac{(\Phi_1 + \Phi_2)}{2} \tag{6}$$

Dielectric relaxation analysis

Modified Curie-Weiss Law was used to fit the dielectric permittivity of CST compositions at 100 kHz, given by:

$$\frac{1}{\varepsilon} = \frac{1}{\varepsilon_m} + \frac{(T - T_m)^\gamma}{C} \tag{7}$$

where C is the Curie-Weiss constant and the exponent γ, ranging between 1 and 2, characterizes the degree of diffuseness. A γ value near 1 indicates a normal ferroelectric, while a value close to 2 suggests an ideal relaxor ferroelectric (*49*).

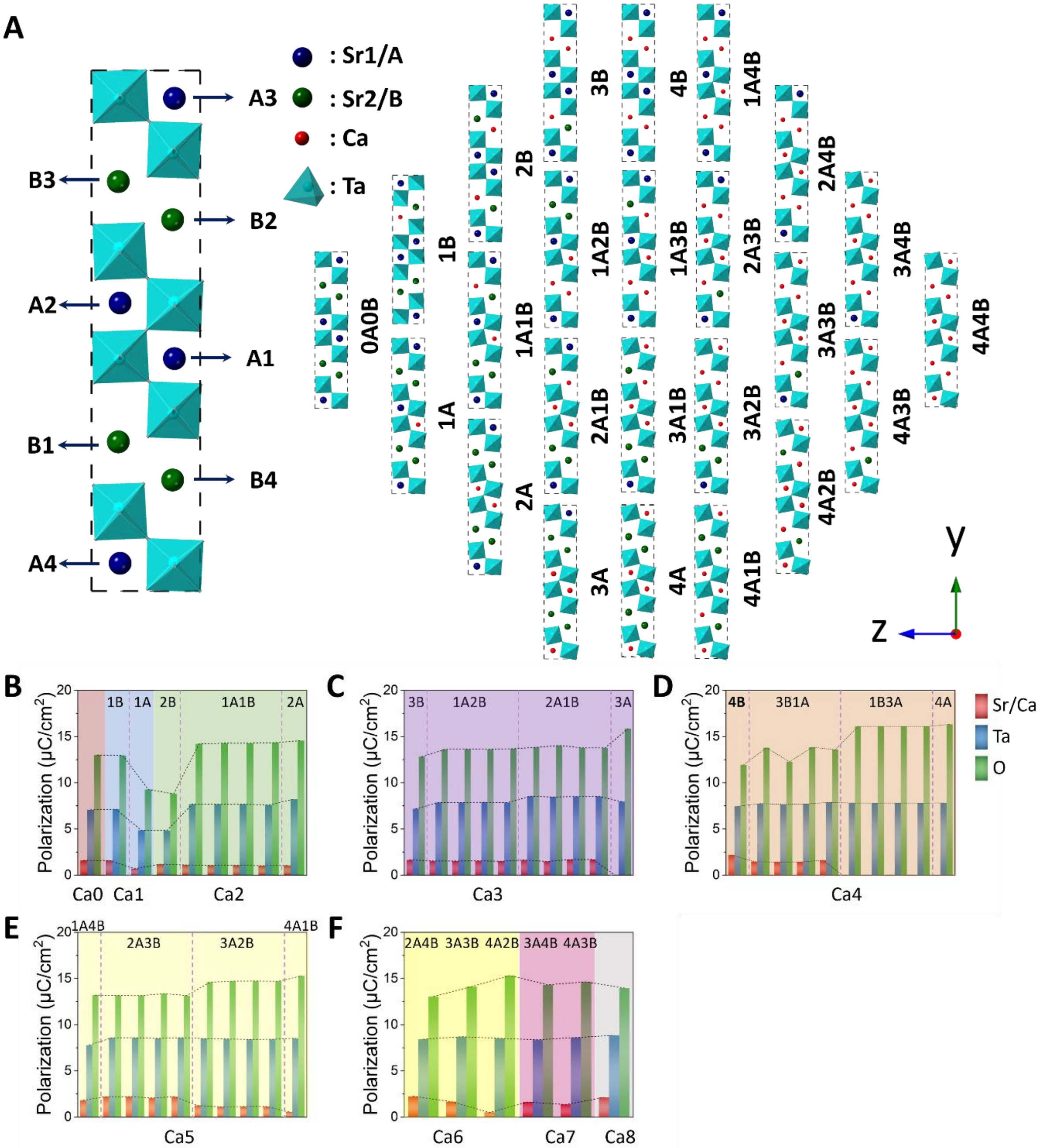

Fig. S1. DFT calculation of the CST system. (**A**) Relaxed crystal structure of $Sr_2Ta_2O_7$ viewed along the a-axis, showing site-dependent Ca substitution within the layered framework. The notation *n*A*m*B denotes the configurations where *n* and *m* Ca atoms occupy the A-site (Sr1) and B-site (Sr2) positions, respectively. (**B-F**) Elemental polarization values for Sr/Ca, Ta, and O, calculated from off-center displacements and Born effective charges obtained from DFT simulation for $Sr_{8-x}Ca_xTa_8O_{28}$ (Ca*x*) compositions: (**B**) $x = 0, 1, 2$; (**C**) $x = 3$; (**D**) $x = 4$; (**E**) $x = 5$; and (**F**) $x = 6, 7, 8$.

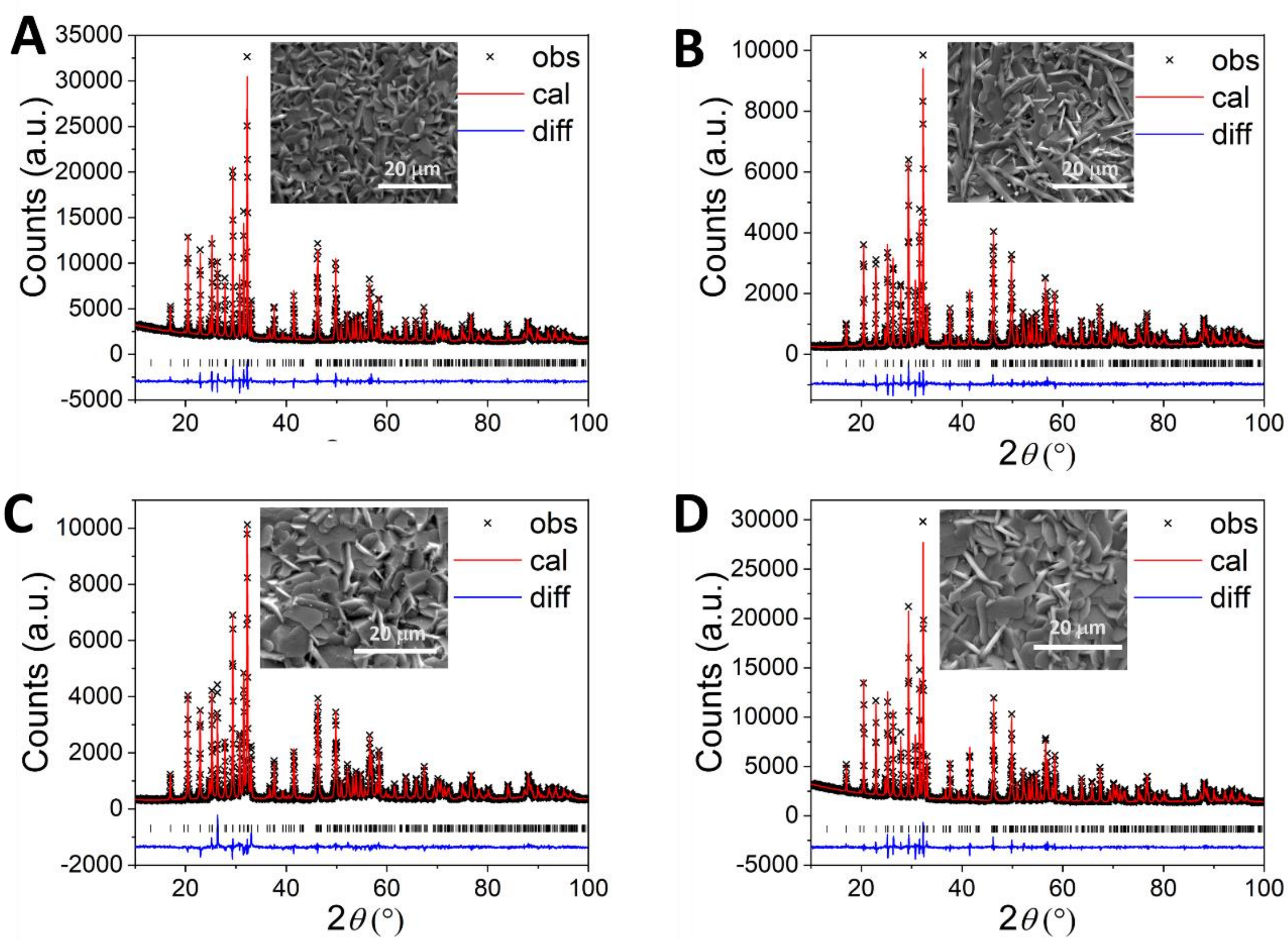

Fig. S2. Rietveld refinements of CST powder samples. Fitted XRD patterns and corresponding surface morphologies shown in SEM insets for (**A**) CST06, (**B**) CST08, (**C**) CST10, and (**D**) CST12.

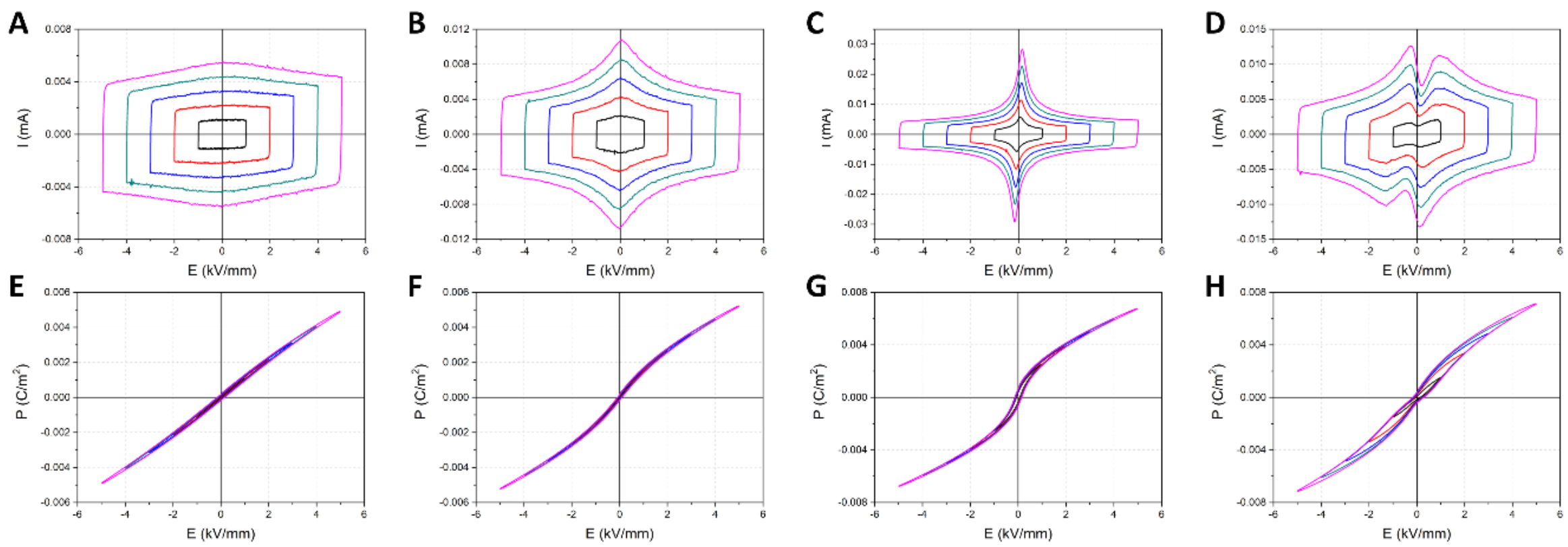

Fig. S3. Ferroelectric measurements at 10 Hz. *I-E* and *P-E* loops measured at different electric fields for (**A, E**) CST06, (**B, F**) CST08, (**C, G**) CST10 and (**D, H**) CST12 compositions.

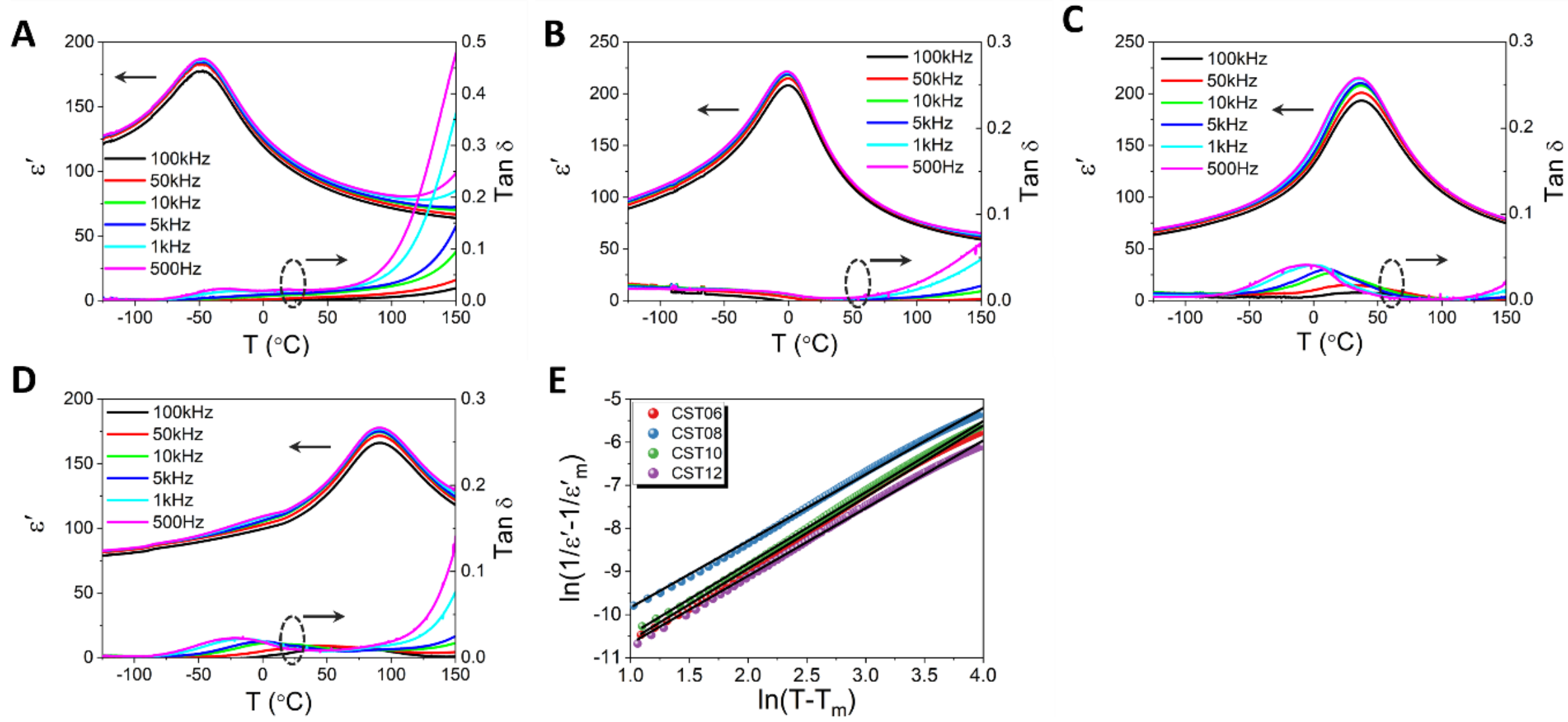

Fig. S4. Temperature dependence of dielectric permittivity and loss for studied compositions, (**A**) CST06, (**B**) CST08, (**C**) CST10 and (**D**) CST12 compositions. (**E**) Modified Curie-Weiss fits to the dielectric permittivity data.

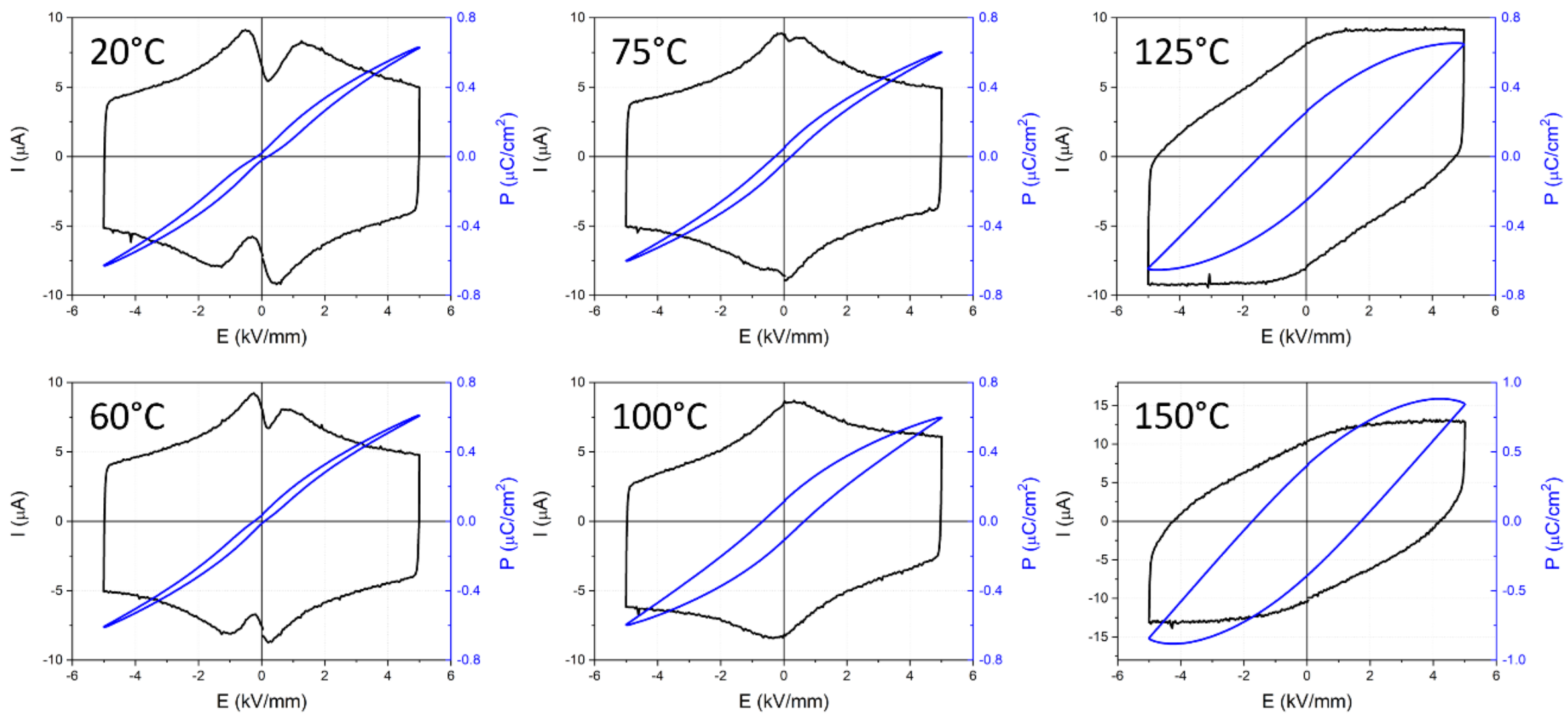

Fig. S5. Temperature-dependent *P-E* and *I-E* loops for CST12.

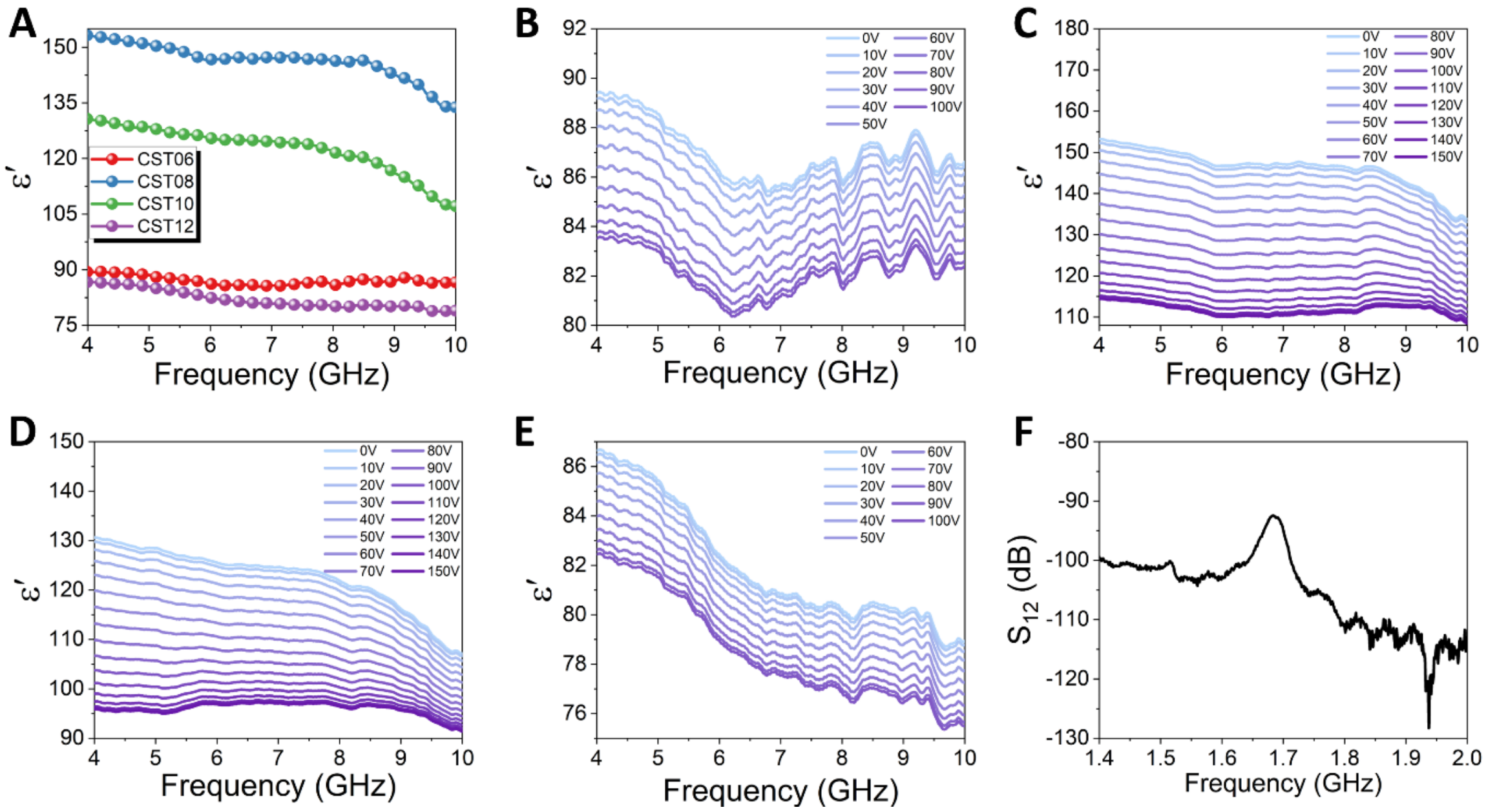

Fig. S6. Microwave dielectric response of the studied compositions. (A) Microwave frequency dependence of dielectric permittivity. Frequency dependence of dielectric permittivity under different applied DC bias fields for (**B**) CST06, (**C**) CST08, (**D**) CST10 and (**E**) CST12. (**F**) Frequency dependence of transmission spectrum (S_{12}) measured using the resonant cavity method.

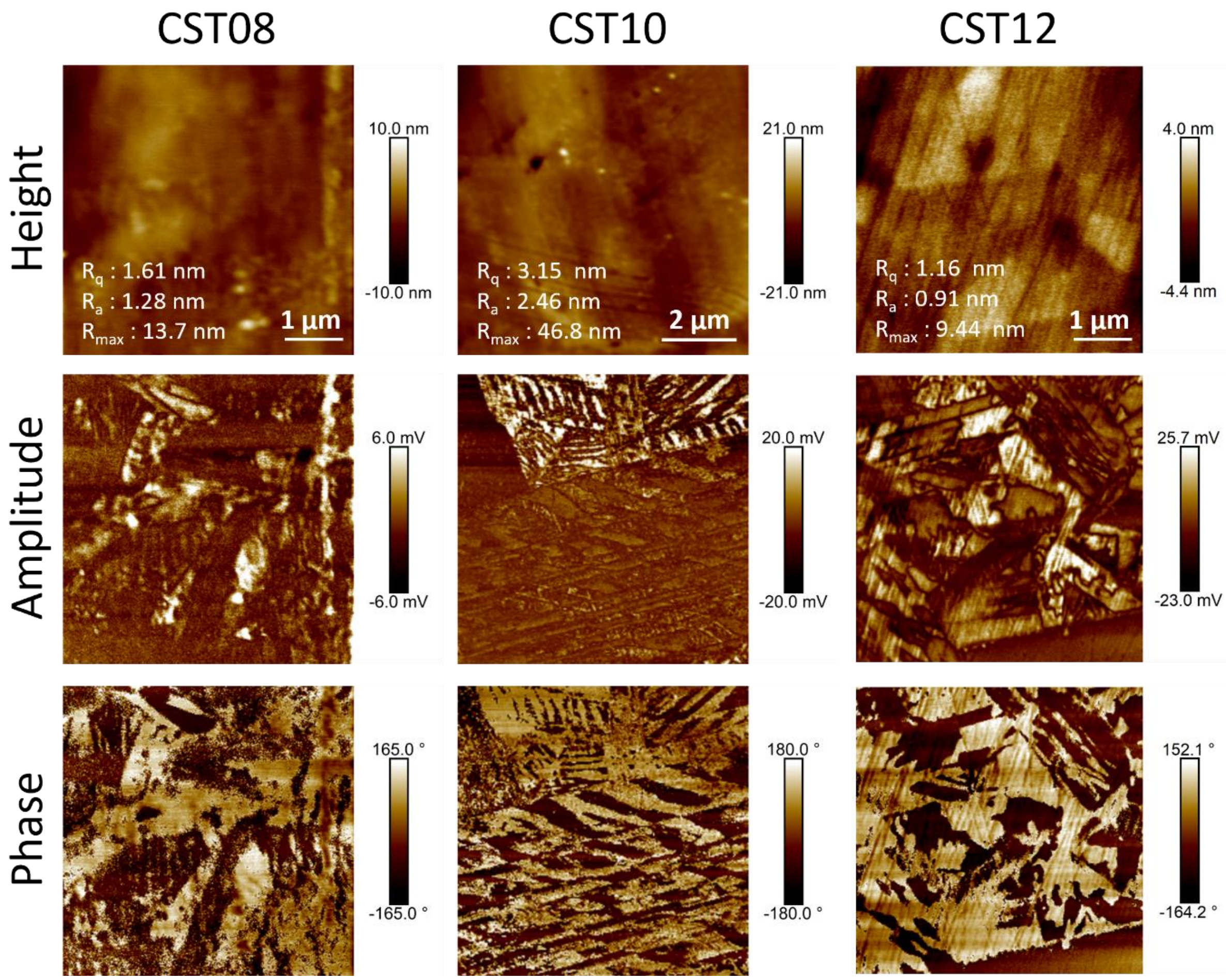

Fig. S7. PFM images of CST08, CST10 and CST12.

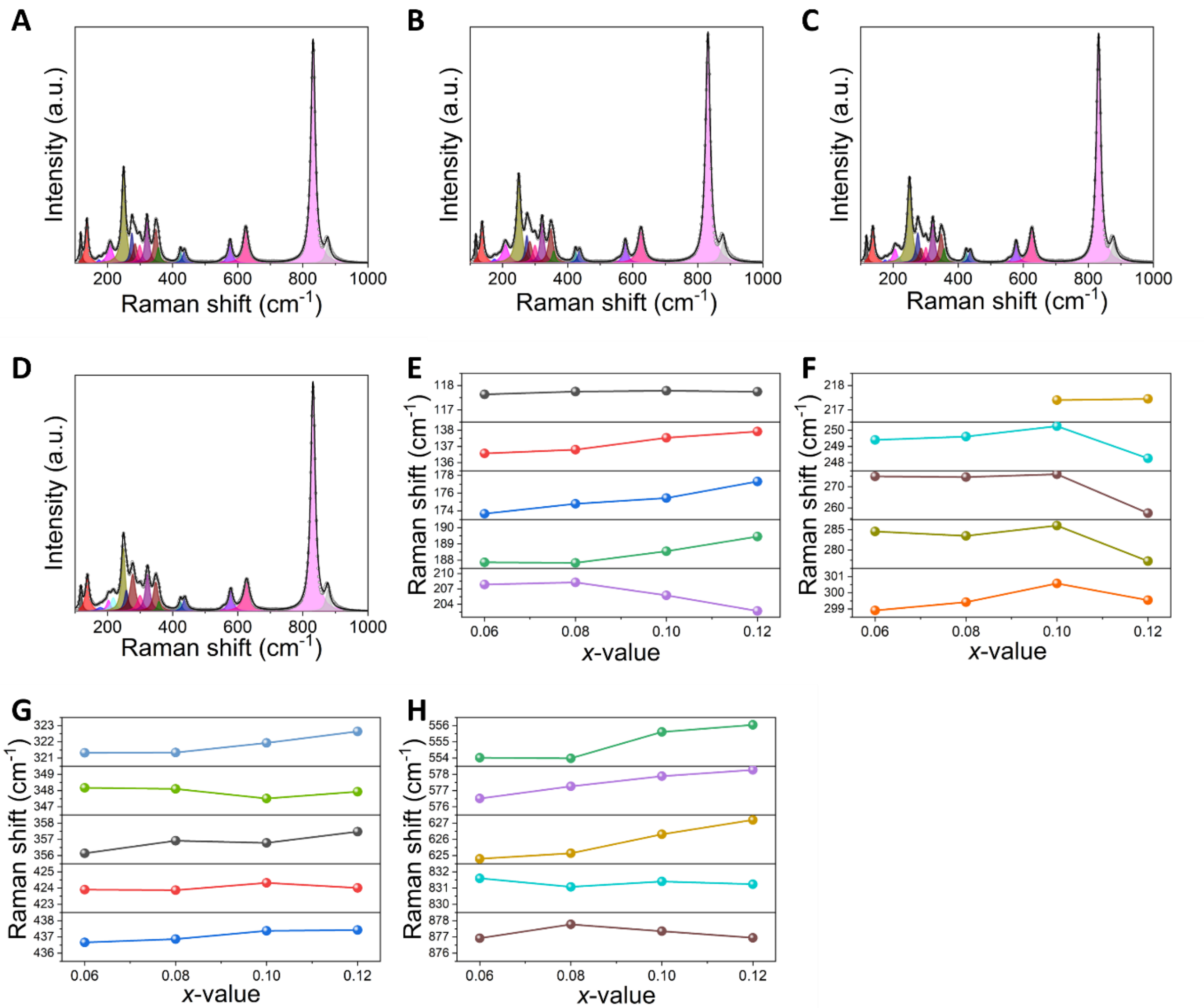

Fig. S8. Raman spectral analysis of the studied compositions. Fitted Raman spectra for (**A**) CST06, (**B**) CST08, (**C**) CST10 and (**D**) CST12 compositions. (**E-H**) Raman peak positions as a function of Ca substitution level (*x*), indicating local structural distortions and modified bonding environments induced by Ca substitution.

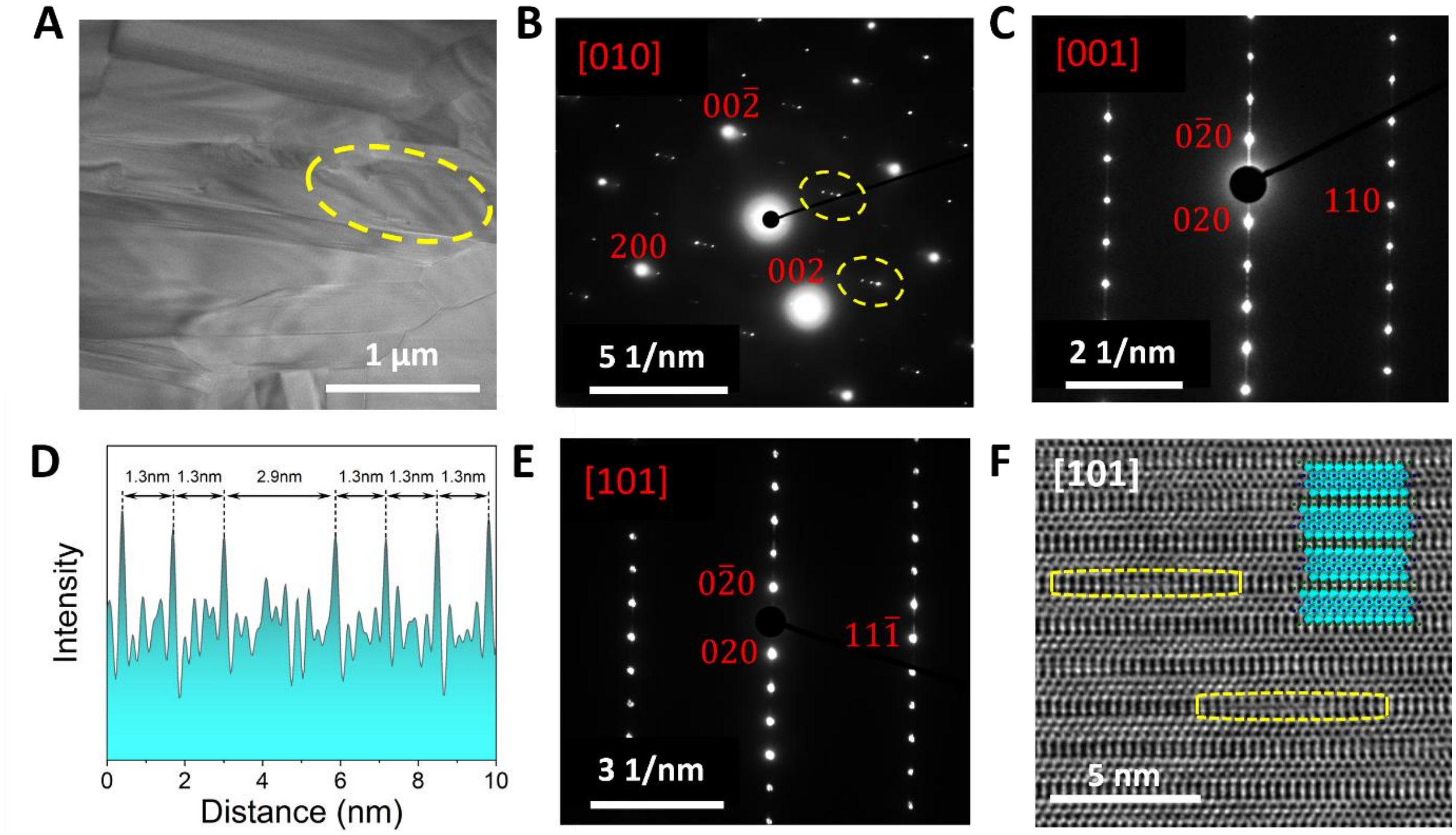

Fig. S9. (A) TEM image of the CST08 composition with stripe patterns (marked by a yellow dashed circle); (**B**) SAED image along [010] direction, with extra diffraction spots (marked by circles); (**C**) SAED image of CST08 along [001]; (**D)** Intensity profile of the rectangular region shown in Fig. 3C; (**E**) SAED image of CST08 along [101]; (**F**) HRTEM image along [101] direction with two defect regions.

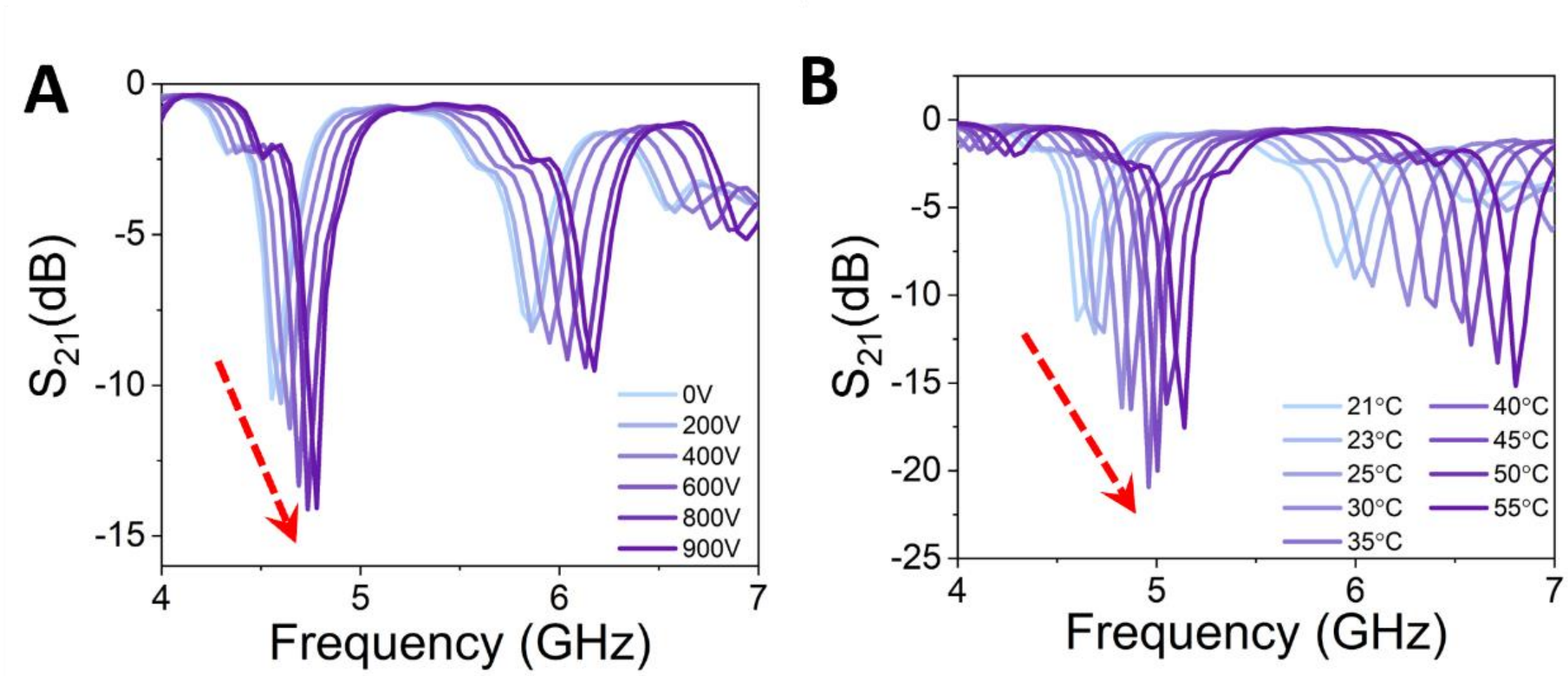

Fig. S10. Transmission spectra (S_{21}) of the microwave resonator measured under different (A) DC voltages and (B) temperatures.

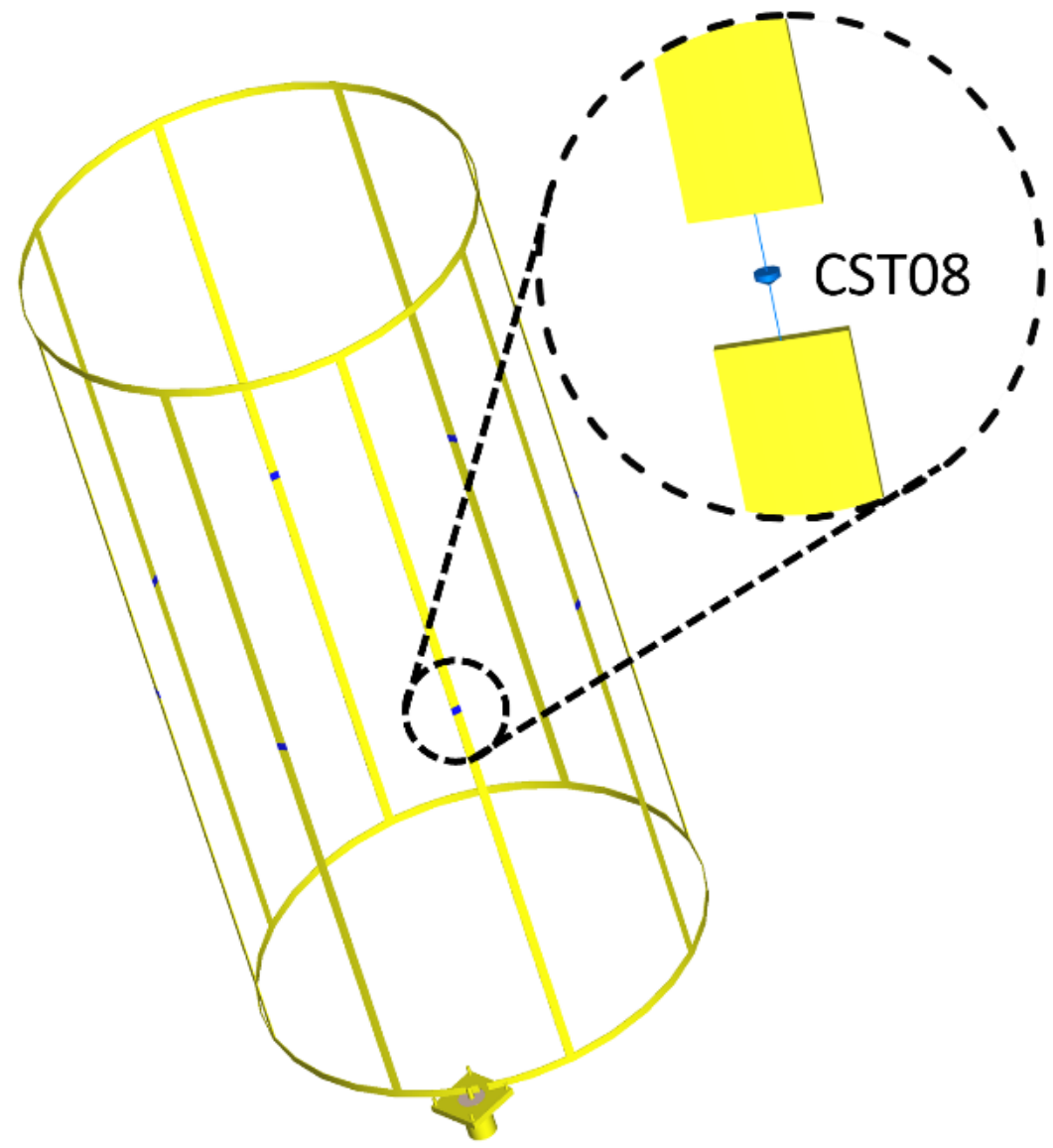

Fig. S11. Schematic of the birdcage antenna integrated with the CST08 ceramic sample.

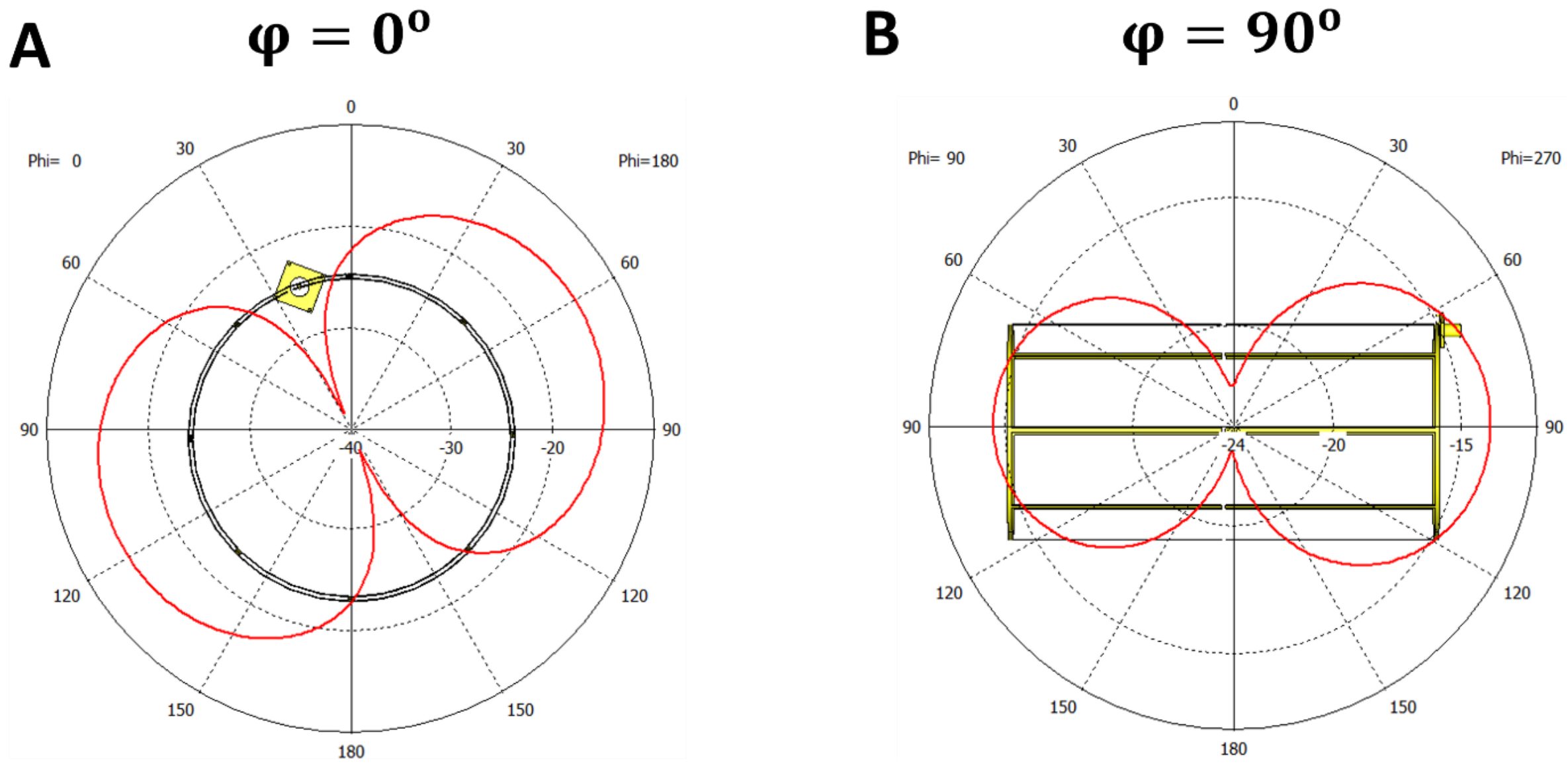

Fig. S12. (A) Simulated far-field radiation pattern in the E-plane, and (B) the corresponding H-plane distribution at the operating frequency.

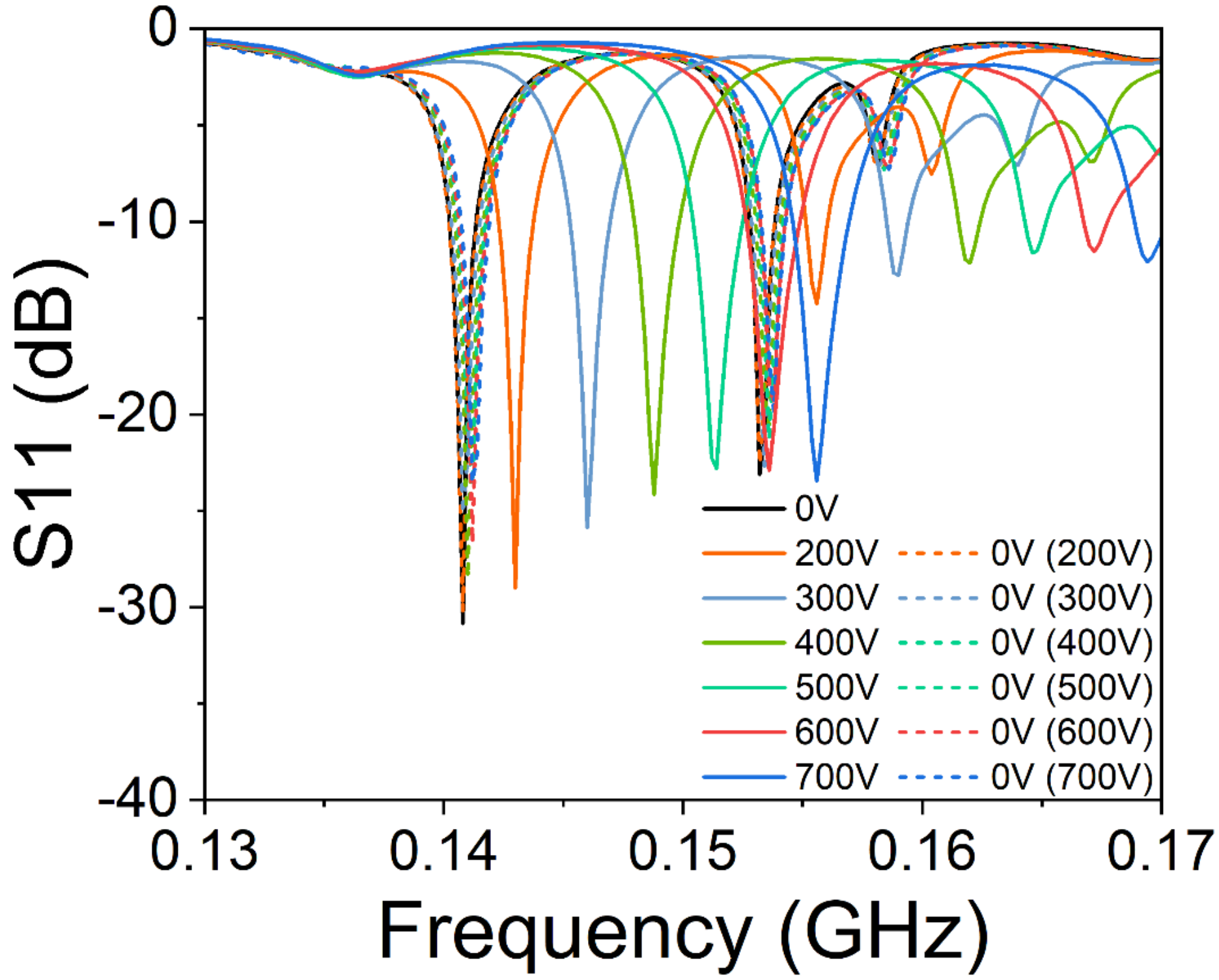

Fig. S13. Bias-on/bias-off repeatability of the CST08 birdcage resonator. Reflection coefficient S_{11} spectra were measured sequentially at 0 V, followed by increasing DC bias voltages from 200 to 700 V. After each biased measurement, the voltage was removed and the S_{11} spectrum was re-measured at 0 V, denoted as 0 V (200 V), 0 V (300 V), etc.

Table S1. Calculated lattice parameters (*a*, *b*, *c*), unit-cell volumes, and TOTEN for $Sr_{8-x}Ca_xTa_8O_{28}$ (Ca*x*) compositions obtained from DFT-relaxed structures.

Composition	Configuration	*a* (Å)	*b* (Å)	*c* (Å)	unit cell volume (Å^3)	TOTEN (eV)
**Ca0**	-	3.964164	27.33308	5.708926	618.5782	-823.42035
**Ca1**	B1	3.954582	27.29083	5.689115	613.9912	-815.22792
	A1	3.964205	27.10431	5.692057	611.5948	-815.06678
**Ca2**	B1, B2	3.943833	27.25518	5.669135	609.3747	-807.04849
	A3, B1	3.953418	27.10319	5.672618	607.8223	-806.88834
	A4, B2	3.95348	27.10012	5.672509	607.7513	-806.88832
	A2, B4	3.953427	27.09912	5.672538	607.7241	-806.88832
	A1, B3	3.953248	27.10148	5.67258	607.7536	-806.88820
	A1, A2	3.957962	26.95222	5.683965	606.3419	-806.86873
**Ca3**	B1, B2, B3	3.933535	27.22976	5.648157	604.9696	-798.92778
	A3, B2, B3	3.942887	27.12893	5.651916	604.5643	-798.71687
	A2, B2, B3	3.942744	27.12736	5.651875	604.5029	-798.71682
	A4, B1, B4	3.942991	27.12817	5.652056	604.5782	-798.71679
	A1, B1, B4	3.943039	27.12452	5.651855	604.4828	-798.71679
	A1, A2, B2	3.947346	26.9705	5.664252	603.0268	-798.67001
	A1, A2, B1	3.947407	26.9641	5.66367	602.831	-798.66995
	A3, A4, B3	3.947024	26.97548	5.664295	603.0935	-798.66991
	A3, A4, B4	3.946881	26.97727	5.66438	603.1205	-798.66988
	A1, A2, A3	3.953286	26.77457	5.669397	600.0917	-798.55817
**Ca4**	B1, B2, B3, B4	3.923292	27.2125	5.627089	600.7625	-790.82496
	A3, B2, B3, B4	3.931895	27.09068	5.631157	599.818	-790.57571
	A2, B1, B2, B3	3.931984	27.09108	5.631185	599.8434	-790.57566
	A1, B1, B2, B4	3.932028	27.08939	5.631333	599.8283	-790.57564
	A4, B1, B3, B4	3.932296	27.09021	5.630817	599.8326	-790.57556
	A1, A2, A4, B1	3.943182	26.76102	5.647309	595.9237	-790.38127
	A2, A3, A4, B3	3.943149	26.76098	5.64721	595.9075	-790.38125
	A1, A3, A4, B4	3.943165	26.76012	5.647235	595.8933	-790.38125
	A1, A2, A3, B2	3.943196	26.75983	5.647086	595.8759	-790.38123
	A1, A2, A3, A4	3.947767	26.60874	5.653774	593.9013	-790.38315
**Ca5**	A1, B1, B2, B3, B4	3.920844	27.08836	5.609191	595.7478	-782.48827
	A3, A4, B2, B3, B4	3.925914	26.97623	5.620048	595.1989	-782.39130
	A3, A4, B1, B3, B4	3.925925	26.97622	5.620034	595.1987	-782.39130
	A1, A2, B1, B2, B3	3.926068	26.96802	5.619771	595.0117	-782.39123
	A1, A2, B1, B2, B4	3.925864	26.97671	5.620228	595.2209	-782.39126
	A1, A3, A4, B1, B4	3.936036	26.81189	5.618965	592.9838	-782.22486
	A2, A3, A4, B2, B3	3.936197	26.80367	5.618425	592.7692	-782.22480
	A1, A2, A3, B2, B3	3.935682	26.80877	5.619168	592.8828	-782.22468
	A1, A2, A4, B1, B4	3.935757	26.81016	5.618996	592.9067	-782.22477
	A1, A2, A3, A4, B1	3.938541	26.62221	5.630686	590.3924	-782.22171
**Ca6**	A1, A2, B1, B2, B3, B4	3.914968	26.97098	5.596378	590.9245	-774.32155
	A1, A2, A3, B1, B2, B3	3.925812	26.79932	5.596576	588.8107	-774.09747
	A1, A2, A3, A4, B1, B2	3.928422	26.63222	5.604988	586.4085	-774.07354

**Ca7**	A1, A2, A3, B1, B2, B3, B4	3.914574	26.80684	5.571707	584.6802	-766.02934
	A1, A2, A3, A4, B1, B2, B3	3.919601	26.65346	5.579112	582.8549	-765.97097
**Ca8**	all sites	3.909325	26.68628	5.55392	579.4145	-757.89393

Table S2. Crystal and refinement parameters obtained from Rietveld fits to the CST compositions. Estimated standard deviations are given in parentheses.

**Chemical formula**	$(Sr_{0.94}Ca_{0.06})_2Ta_2O_7$	$(Sr_{0.92}Ca_{0.08})_2Ta_2O_7$	$(Sr_{0.90}Ca_{0.10})_2Ta_2O_7$	$(Sr_{0.88}Ca_{0.12})_2Ta_2O_7$
**Space group**	*Cmcm*	*Cmcm*	*Cmcm*	$Cmc2_1$
**Unit cell dimensions**	*a* = 3.94135(5) Å<br>*b* = 27.1802(3) Å<br>*c* = 5.68532(7) Å	*a* = 3.93939(6) Å<br>*b* = 27.1680(4) Å<br>*c* = 5.68328(9) Å	*a* = 3.93797(8) Å<br>*b* = 27.1492(6) Å<br>*c* = 5.6777(1) Å	*a* = 3.93553(5) Å<br>*b* = 27.1478(3) Å<br>*c* = 5.67534(7) Å
**Volume (Å^3)**	609.05(1)	608.25(1)	607.02(2)	606.36(1)
**Z**	4	4	4	4
D_{calc} **(g cm^{-3})**	7.017	7.005	6.999	6.986
**R-factors**	R_{wp} = 0.0467<br>R_p = 0.0323<br>χ^2 = 4.920<br>R_F^2 = 0.0676<br>R_{exp} = 0.0211	R_{wp} = 0.0704<br>R_p = 0.0528<br>χ^2 = 2.406<br>R_F^2 = 0.0537<br>R_{exp} = 0.0456	R_{wp} = 0.0737<br>R_p = 0.0530<br>χ^2 = 3.120<br>R_F^2 = 0.0692<br>R_{exp} = 0.0419	R_{wp} = 0.0495<br>R_p = 0.0340<br>χ^2 = 5.334<br>R_F^2 = 0.0758<br>R_{exp} = 0.0215
**No. of variables**	30	30	30	33
**No. of profile points**	3649	3440	3440	3649

Table S3. Parameters obtained from modified Curie-Weiss fits for the CST compositions and their dielectric properties.

Compositions	T_c (°C) @ 100 kHz	γ	C	ε' @ 1 MHz	Tanδ @ 1 MHz
**CST06**	-47.6	1.66	2.1×10^5	110	0.005
**CST08**	1.7	1.54	8.8×10^4	178	0.003
**CST10**	38.4	1.65	1.8×10^5	231	0.018
**CST12**	92.8	1.57	2.1×10^5	100	0.011

Table S4. Comparison of microwave dielectric tunability, loss, and figure of merit (FoM) for tunable dielectrics.

Material system	ε'	tan δ	Frequency (GHz)	Tunability (%)	Bias field (kV/cm)	FoM*	Reference
**$Ba_{0.5}Sr_{0.5}TiO_3$**	1622	0.03	~7.7	22	8.1	7.3	Chong *et al.*, 2004 (*50*)
**$Ba_{0.5}Sr_{0.5}TiO_3$-40%Al_2O_3**	870	0.011	~7.7	15.9	8.1	14.5	Chong *et al.*, 2004 (*50*)
**$Ba_{0.5}Sr_{0.5}TiO_3$-$Bi_{1.5}Zn_{1.0}Nb_{1.5}O_7$**	471	0.0048	~7.7	6.2	8.1	12.9	Yan *et al.*, 2004 (*51*)
**$AgTa_{0.1}Nb_{0.9}O_3$**	~160	0.15	4	~5	10	0.3	Zimmermann *et al.*, 2004 (*52*)
**$Sr_6BaTi_6O_{19}$**	350	0.018	10	46	400	25.6	Dawley *et al.*, 2020 (*3*)
**$Pb_{0.4}Sr_{0.6}Ti_{0.97}Mn_{0.03}O_3$**	~1550	~0.1	10	35	40	3.5	Liu *et al.*, 2005 (*53*)
**$Ba_{0.6}Sr_{0.4}TiO_3$**	~500	0.01	8	20	200	20.0	Chang *et al.*, 2002 (*54*)
**$(Ca_{0.08}Sr_{0.92})_2Ta_2O_7$**	172.7	0.009	1.68	24	30	26.7	This work

*Note: FoM = Tunability / tan δ

REFERENCES

1. J. H. Lee, G. Luo, I. C. Tung, S. H. Chang, Z. Luo, M. Malshe, M. Gadre, A. Bhattacharya, S. M. Nakhmanson, J. A. Eastman, H. Hong, J. Jellinek, D. Morgan, D. D. Fong, J. W. Freeland, Dynamic layer rearrangement during growth of layered oxide films by molecular beam epitaxy. *Nat. Mater.* **13**, 879–883 (2014).

2. X. Z. Lu, J. M. Rondinelli, Epitaxial-strain-induced polar-to-nonpolar transitions in layered oxides. *Nat. Mater.* **15**, 951–955 (2016).

3. N. M. Dawley, E. J. Marksz, A. M. Hagerstrom, G. H. Olsen, M. E. Holtz, V. Goian, C. Kadlec, J. Zhang, X. Lu, J. A. Drisko, R. Uecker, S. Ganschow, C. J. Long, J. C. Booth, S. Kamba, C. J. Fennie, D. A. Muller, N. D. Orloff, D. G. Schlom, Targeted chemical pressure yields tuneable millimetre-wave dielectric. *Nat. Mater.* **19**, 176–181 (2020).

4. X. Xiong, H. Liu, J. Zhang, L. L. da Silva, Z. Sheng, Y. Yao, G. Wang, M. Hinterstein, S. Zhang, J. Chen, Ultrahigh energy-storage in dual-phase relaxor ferroelectric ceramics. *Adv. Mater.* **36**, e2410088 (2024).

5. F. Li, B. Wang, X. Gao, D. Damjanovic, L.-Q. Chen, S. Zhang, Ferroelectric materials toward next-generation electromechanical technologies. *Science* **389**, eadn4926 (2025).

6. W. Li, X. Zhang, J. Yang, S. Zhou, C. Song, P. Cheng, Y.-Q. Zhang, B. Feng, Z. Wang, Y. Lu, K. Wu, L. Chen, Emergence of ferroelectricity in a nonferroelectric monolayer. *Nat. Commun.* **14**, 2757 (2023).

7. E. Bousquet, M. Dawber, N. Stucki, C. Lichtensteiger, P. Hermet, S. Gariglio, J. M. Triscone, P. Ghosez, Improper ferroelectricity in perovskite oxide artificial superlattices. *Nature* **452**, 732–736 (2008).

8. W. Cao, T. Li, K. Li, Y. Huang, H. Xie, Y. Yao, Z. Sun, C. Lou, W. Zhang, C. Xu, L. Zhu, B. Xie, J. Zhang, M. G. Tucker, H. Liu, H. Luo, M. Tang, J. Chen, Unleashed remarkable energy storage performance in $Bi_{0.5}K_{0.5}TiO_3$-based relaxor ferroelectrics by local structural fluctuation. *Angew. Chem. Int. Ed. Engl.* **64**, e202416291 (2025).

9. Y. S. Oh, X. Luo, F. T. Huang, Y. Wang, S. W. Cheong, Experimental demonstration of hybrid improper ferroelectricity and the presence of abundant charged walls in $(Ca,Sr)_3Ti_2O_7$ crystals. *Nat. Mater.* **14**, 407–413 (2015).

10. Z. Xu, Y. Liu, M. Azadeh, H. Thong, Y. Jiang, F. Yao, Z. Yue, Z. Zhang, Z. Tang, J. Li, H. Wang, T. Frömling, K. Wang, Identifying the Interfacial Polarization in Non-stoichiometric Lead-Free Perovskites by Defect Engineering. *Angew. Chem. Int. Ed. Engl.* **62**, e202216776 (2023).

11. J. Costantine, Y. Tawk, S. E. Barbin, C. G. Christodoulou, Reconfigurable antennas: Design and applications. *Proc. IEEE* **103**, 424–437 (2015).

12. X. Chou, J. Zhai, X. Yao, Dielectric tunable properties of low dielectric constant $Ba_{0.5}Sr_{0.5}TiO_3$-Mg_2TiO_4 microwave composite ceramics. *Appl. Phys. Lett.* **91**, 122908 (2007).

13. Q. Luo, S. Gao, Y. Wang, I. Bakaimi, A. Mostaed, C. K. de Groot, B. Hayden, I. Reaney, "Electronically Beam-steerable Dual-band Reflectarray for Satellite Communications" in *2019 IEEE-APS Topical Conference on Antennas and Propagation in Wireless Communications (APWC)* (IEEE, 2019), pp. 050–055, https://ieeexplore.ieee.org/document/8870431/.

14. M. Zhang, J. Zhai, X. Yao, Microwave dielectric properties of high dielectric tunable - Low permittivity $Ba_{0.5}Sr_{0.5}TiO_3$-$Mg_2(Ti_{0.95}Sn_{0.05})O_4$ composite ceramics. *Ceram. Int.* **38**, S173–S176 (2012).

15. A. Ahmed, I. A. Goldthorpe, A. K. Khandani, Electrically tunable materials for microwave applications. *Appl. Phys. Rev.* **2**, 011302 (2015).

16. L. B. Kong, S. Li, T. S. Zhang, J. W. Zhai, F. Y. C. Boey, J. Ma, Electrically tunable dielectric materials and strategies to improve their performances. *Prog. Mater. Sci.* **55**, 840–893 (2010).

17. A. Feteira, D. C. Sinclair, I. M. Reaney, Y. Somiya, M. T. Lanagan, $BaTiO_3$-based ceramics for tunable microwave applications. *J. Am. Ceram. Soc.* **87**, 1082–1087 (2004).

18. H. Zhang, H. Giddens, Y. Yue, X. Xu, V. Araullo-Peters, V. Koval, M. Palma, I. Abrahams, H. Yan, Y. Hao, Polar nano-clusters in nominally paraelectric ceramics demonstrating high microwave tunability for wireless communication. *J. Eur. Ceram. Soc.* **40**, 3996–4003 (2020).

19. C. Mao, S. Yan, C. Yao, F. Cao, G. Wang, X. Dong, X. Meng, Degraded grain size effect of barium strontium titanate ceramics under a direct current bias electric field. *Mater. Res. Express* **4**, 016302 (2017).

20. M. Acosta, N. Novak, V. Rojas, S. Patel, R. Vaish, J. Koruza, G. A. Rossetti, J. Rödel, $BaTiO_3$-based piezoelectrics: Fundamentals, current status, and perspectives. *Appl. Phys. Rev.* **4**, 041305 (2017).

21. X. Wei, Y. Feng, L. Hang, X. Yao, Dielectric properties of barium stannate titanate ceramics under bias field. *Ceram. Int.* **30**, 1401–1404 (2004).

22. X.-X. Huang, T.-F. Zhang, X.-G. Tang, Y.-P. Jiang, Q.-X. Liu, Z.-Y. Feng, Q.-F. Zhou, Dielectric relaxation and pinning phenomenon of $(Sr,Pb)TiO_3$ ceramics for dielectric tunable device application. *Sci. Rep.* **6**, 31960 (2016).

23. J. P. Turpin, J. A. Bossard, K. L. Morgan, D. H. Werner, P. L. Werner, Reconfigurable and Tunable Metamaterials: A Review of the Theory and Applications. *Int. J. Antennas Propag.* **2014**, 429837 (2014).

24. N. Ishizawa, F. Marumo, T. Kawamura, M. Kimura, Compounds with perovskite-type slabs. II. The crystal structure of $Sr_2Ta_2O_7$. *Acta Crystallogr. Sect. B Struct. Crystallogr. Cryst. Chem.* **32**, 2564–2566 (1976).

25. N. Yamamoto, K. Yagi, G. Honjo, M. Kimura, T. Kawamura, New phases of $Sr_2Ta_2O_7$ and $Sr_2Nb_2O_7$ found by electron microscopy and diffraction. *J. Physical Soc. Japan* **48**, 185–191 (1980).

26. N. Ishizawa, F. Marumo, S. Iwai, Compounds with perovskite-type slabs. IV. Ferroelectric phase transitions in $Sr_2(Ta_{1-x}Nb_x)_2O_7$ ($x \simeq 0.12$) and $Sr_2Ta_2O_7$. *Acta Crystallogr. Sect. B Struct. Crystallogr. Cryst. Chem.* **37**, 26–31 (1981).

27. H. Zhang, H. Gidden, T. G. Saunders, N. Liu, V. Aurallo-Peters, X. Xu, M. Palma, M. J. Reece, I. Abrahams, H. Yan, Y. Hao, High tunability and low loss in layered perovskite dielectrics through intrinsic elimination of oxygen vacancies. *Chem. Mater.* **32**, 10120–10129 (2020).

28. H. Zhang, A. D. Fortes, H. Giddens, T. G. Saunders, M. Palma, I. Abrahams, H. Yan, Y. Hao, Antiferroelectric-like behavior in a lead-free perovskite layered structure ceramic. *Inorg. Chem.* **61**, 20316–20325 (2022).

29. M. J. Pitcher, P. Mandal, M. S. Dyer, J. Alaria, P. Borisov, H. Niu, J. B. Claridge, M. J. Rosseinsky, Tilt engineering of spontaneous polarization and magnetization above 300 K in a bulk layered perovskite. *Science* **347**, 420–424 (2015).

30. N. A. Benedek, C. J. Fennie, Hybrid improper ferroelectricity: A mechanism for controllable polarization-magnetization coupling. *Phys. Rev. Lett.* **106**, 107204 (2011).

31. C. Chen, Q. Jiang, X. Wei, I. Abrahams, H. Yan, M. J. Reece, Three layer perovskite-like structured $Pr_3Ti_2TaO_{11}$ ferroelectrics with super-high curie point. *J. Am. Ceram. Soc.* **97**, 3624–3630 (2014).

32. H. Yan, H. Ning, Y. Kan, P. Wang, M. J. Reece, Piezoelectric ceramics with super-high curie points. *J. Am. Ceram. Soc.* **92**, 2270–2275 (2009).

33. P. A. Fuierer, T. R. Shrout, R. E. Newnham, Physical, electrical, and piezoelectric properties of hot-forged $Sr_2(NbTa)_2O_7$ ceramics. *MRS Online Proc. Libr.* **276**, 51–57 (1992).

34. J.-S. Zhou, J. B. Goodenough, Chemical bonding and electronic structure of $R\mathrm{NiO}_3$ (R = rare earth). *Phys. Rev. B* **69**, 153105 (2004).

35. F. Shao, C. Chen, X. He, L. Wang, M. A. Boda, Z. Yi, Textured $Sr_2Nb_2O_7$ ceramics: Microstructure design, high temperature ferroelectric and piezoelectric performance. *J. Am. Ceram. Soc.* **108**, e20120 (2025).

36. P. Ghosez, J.-P. Michenaud, X. Gonze, Dynamical atomic charges: The case of ABO_3 compounds. *Phys. Rev. B* **58**, 6224–6240 (1998).

37. R. D. Shannon, Revised effective ionic radii and systematic studies of interatomic distances in halides and chalcogenides. *Acta Crystallogr. Sect. A* **32**, 751–767 (1976).

38. A. K. Tagantsev, V. O. Sherman, K. F. Astafiev, J. Venkatesh, N. Setter, Ferroelectric materials for microwave tunable applications. *J. Electroceram.* **11**, 5–66 (2003).

39. A. Bencan, E. Oveisi, S. Hashemizadeh, V. K. Veerapandiyan, T. Hoshina, T. Rojac, M. Deluca, G. Drazic, D. Damjanovic, Atomic scale symmetry and polar nanoclusters in the paraelectric phase of ferroelectric materials. *Nat. Commun.* **12**, 3509 (2021).

40. H. Fujimori, I. Kawanishi, N. Matsushita, *In situ* Observation of 15 mol% Ba-Substituted Strontium Tantalate up to 1773 K by Ultraviolet Laser Raman Spectroscopy. *Trans. Mater. Res. Soc. Japan* **43**, 7–12 (2018).

41. N. Yamamoto, K. Ishizuka, Analysis of the incommensurate structure in $Sr_2Nb_2O_7$ by electron microscopy and convergent-beam electron diffraction. *Acta Crystallogr. Sect. B Struct. Sci.* **39**, 210–216 (1983).

42. A. C. Larson, R. B. Von Dreele, *General Structure Analysis System (GSAS)*, Los Alamos National Laboratory Report LAUR 86-748 (2004).

43. G. Viola, T. Saunders, X. Wei, K. B. Chong, H. Luo, M. J. Reece, H. Yan, Contribution of piezoelectric effect, electrostriction and ferroelectric/ferroelastic switching to strain-electric field response of dielectrics. *J. Adv. Dielectr.* **03**, 1350007 (2013).

44. G. Kresse, J. Hafner, Ab initio molecular dynamics for liquid metals. *Phys. Rev. B* **47**, 558–561 (1993).

45. J. W. Furness, A. D. Kaplan, J. Ning, J. P. Perdew, J. Sun, Accurate and numerically efficient r^2SCAN meta-generalized gradient approximation. *J. Phys. Chem. Lett.* **11**, 8208–8215 (2020).

46. J. P. Perdew, K. Burke, M. Ernzerhof, Generalized gradient approximation made simple. *Phys. Rev. Lett.* **77**, 3865–3868 (1996).

47. P. E. Blöchl, Projector augmented-wave method. *Phys. Rev. B* **50**, 17953–17979 (1994).

48. K. Robinson, G. V. Gibbs, P. H. Ribbe, Quadratic elongation: A quantitative measure of distortion in coordination polyhedra. *Science* **172**, 567–570 (1971).

49. K. Uchino, S. Nomura, Critical exponents of the dielectric constants in diffused-phase-transition crystals. *Ferroelectrics* **44**, 55–61 (1982).

50. K. B. Chong, L. B. Kong, L. Chen, L. Yan, C. Y. Tan, T. Yang, C. K. Ong, T. Osipowicz, Improvement of dielectric loss tangent of Al_2O_3 doped $Ba_{0.5}Sr_{0.5}TiO_3$ thin films for tunable microwave devices. *J. Appl. Phys.* **95**, 1416–1419 (2004).

51. L. Yan, L. B. Kong, L. F. Chen, K. B. Chong, C. Y. Tan, C. K. Ong, $Ba_{0.5}Sr_{0.5}TiO_3$–$Bi_{1.5}Zn_{1.0}Nb_{1.5}O_7$ composite thin films with promising microwave dielectric properties for microwave device applications. *Appl. Phys. Lett.* **85**, 3522–3524 (2004).

52. F. Zimmermann, W. Menesklou, E. Ivers-Tiffée, Investigation of $Ag(Ta,Nb)O_3$ as tunable microwave dielectric. *J. Eur. Ceram. Soc.* **24**, 1811–1814 (2004).

53. S. W. Liu, J. Weaver, Z. Yuan, W. Donner, C. L. Chen, J. C. Jiang, E. I. Meletis, W. Chang, S. W. Kirchoefer, J. Horwitz, A. Bhalla, Ferroelectric $(Pb,Sr)TiO_3$ epitaxial thin films on (001) MgO for room temperature high-frequency tunable microwave elements. *Appl. Phys. Lett.* **87**, 142905 (2005).

54. W. Chang, S. W. Kirchoefer, J. M. Pond, J. S. Horwitz, L. Sengupta, Strain-relieved $Ba_{0.6}Sr_{0.4}TiO_3$ thin films for tunable microwave applications. *J. Appl. Phys.* **92**, 1528–1535 (2002).
